# Supplementary material for: Leading countries in global science increasingly receive more citations than other countries doing similar research
Source: Nat Hum Behav. 2022 May 30;6(7):919–29. doi: 10.1038/s41562-022-01351-5 (PMC9314251; doi:10.1038/s41562-022-01351-5)
Supplement: Supplementary file 1 — Supplemental Figures S1-S31, Supplemental Tables S1-S4. [file 41562_2022_1351_MOESM1_ESM.pdf]

nature portfolio

https://doi.org/10.1038/s41562-022-01351-5

Supplementary information

Leading countries in global science increasingly receive more citations than other countries doing similar research

In the format provided by the
authors and unedited

Supplementary Information

Title

Leading countries in global science increasingly receive more citations than other countries doing similar research.

Author List

Charles J. Gomez[1]*

Andrew C. Herman[2]

Paolo Parigi[3]

*Corresponding Author

Affiliations

1. Department of Sociology
   Queens College, The City University of New York
   charles.gomez@qc.cuny.edu

2. Department of Sociology
   University of California, Los Angeles
   andrewcherman@ucla.edu

3. Facebook, Inc.
   Department of Civil and Environmental Engineering
   Stanford University
   pparigi@stanford.edu

Nation-Labeled Cohesion Score

Since not every country may produce enough published papers in a year to produce meaningful national signatures, we apply the *umass* topic cohesion measure applied to the nation-labels in each NL-LDA model. For each yearly mode, we compare the document cooccurrences of each nation-label's top 25 strongest associated terms from its national signature. Nation-labels with strong national signatures lead the way in global science and have lexical usage that is more widespread throughout the field than not. Since these measures are unique to each model and not readily comparable across models, we convert each national signature's cohesion score from a given $Field_t$ NL-LDA model into percentile ranks. The nation-labels that are the most ubiquitous like the U.S. and in later years China are in the highest percentile, exhibiting lower coherence scores, and less active countries are in the lowest percentile with higher coherence scores. We rerun Figures 2, 3, and 4 presented in the main paper at the 25th and 75th percentile. Our results broadly hold despite the exclusion of nation-labels.

- Supplementary Figure 1 corresponds to Figure 2A.
- Supplementary Figure 2 corresponds to Figure 2B.
- Supplementary Figure 3 corresponds to Figure 2C.
- Supplementary Figure 4 corresponds to Figure 3A.
- Supplementary Figure 5 corresponds to Figure 3B.
- Supplementary Figure 6 corresponds to Figure 4A.
- Supplementary Figure 7 corresponds to Figure 4B.

Core, Periphery, and Core+Periphery Country Inclusion in the QAP Models

We rerun the QAP models to now include countries beta coefficients that reflect countries at the periphery of scientific communities. In other words, we include a network that only contains peripheral countries, just like the core network only contains core countries.

- Supplementary Figure 8 corresponds to Figure 2B.
- Supplementary Figure 9 corresponds to Figure 2C.

Citation Deflation

A source of bias is the artificial growth of citations due to growing volume of papers. We have implemented two versions of citation deflation based on Petersen, et al.'s work on citation inflation: (1) the field-level measure from Petersen, et al. and (2) a country-field-level measure that extends Petersen, et al.'s field-level approach. On the former, and unless otherwise states, all of our results and fields are field-deflated. On the latter, we calculate the total number of papers that country i has at least one authorship on for each $Field_t$. Much like Petersen et al.'s ratio construction for the total number of papers in the field, we instead deflate at the dyadic level based on the receiver's number of papers in year t to a baseline year. So, if we are looking at the number of citations that the United States received from China, we deflate this number based on the receiver (i.e., the United States) and its number of papers in year t to the baseline year. After all the dyads are deflated, we then calculate the indegree centralities for each receiver using the deflated edge weights. For both field-level and our country-field-level deflation measures, we pick the year 2000 as our baseline year that we choose to both inflate (prior to 2000) and deflate (after 2000) citations. Here, we compare these three treatments (field deflated, country deflated, and no deflation) for Figures 2(B and C), 3, and 4. We find that our conclusions generally hold.

- Supplementary Figure 10 corresponds to Figure 2B.
- Supplementary Figure 11 corresponds to Figure 2C.
- Supplementary Figure 12 corresponds to Figure 3A.
- Supplementary Figure 13 corresponds to Figure 3B.
- Supplementary Figure 14 corresponds to Figure 4A.
- Supplementary Figure 15 corresponds to Figure 4B.

Journal Censoring

A source of bias in the MAG database is the growing population of journals in MAG that could partly drive the increase in country representation, as seen in Figure 2A. If MAG included a smaller set of high impact journals in the early part of the data (i.e., 1980s) with an increasing number of low impact and non-English journals included over time, this may artificially be driving our results. As such, we re-construct Figures 2(B and C), 3, and 4 with journals that are censored since the 1980s (i.e., journals in the data that have curated data each year since 1980 for each field) and with an uncensored population of journals. Unless otherwise stated, all our figures are built using journal censored data. Whether we censor or not, our main findings still hold.

- Supplementary Figure 16 corresponds to Figure 2B.
- Supplementary Figure 17 corresponds to Figure 2C.
- Supplementary Figure 18 corresponds to Figure 3A.
- Supplementary Figure 19 corresponds to Figure 3B.
- Supplementary Figure 20 corresponds to Figure 4A.
- Supplementary Figure 21 corresponds to Figure 4B.

Language Censoring

English is the lingua franca of scientific research, however the inclusion or exclusion of papers whose texts were originally translated into English may bias our findings. In particular, this bias may be introduced when constructing the text similarity network, as the inclusion of these journals may be skewed towards more recent years and potentially distort the growth seen in many of the figures. In the main text, we restricted our abstracts used to build our NL-LDA models and citation networks to English-only abstracts. Here, we re-run our analyses as presented in Figures 2(B and C), 3, and 4 comparing the English-abstract construction with those that include both English abstracts and those translated into English using Google Translate. However, the latter was constructed on an unrestricted and an uncensored version of the data and models, where the citation networks were constructed not using the cites received window, citational deflation, or journal censoring, so the comparison is not entirely equivalent. Nevertheless, our findings remain consistent.

- Supplementary Figure 22 corresponds to Figure 2B.
- Supplementary Figure 23 corresponds to Figure 2C.
- Supplementary Figure 24 corresponds to Figure 3A.
- Supplementary Figure 25 corresponds to Figure 3B.
- Supplementary Figure 26 corresponds to Figure 4A.
- Supplementary Figure 27 corresponds to Figure 4B.

First Appearance of Countries Censoring

For every discipline, we find the year that each country first appeared in the data. Then, we replot Figure 3B and 4B censoring on countries whose first appearances are in different time windows—countries that first appeared in each field between 1980 and 1984, 1980 to 1989, 1980 to 1994, 1980 to 1999, 1980 to 2004, and 1980 to 2012—and recalculate the averages. We find that our results hold.

- Supplementary Figure 28 corresponds to Figure 3B.
- Supplementary Figure 29 corresponds to Figure 4B.

Pooling Statistically Significant and Not Significant Beta Coefficients in the QAP Models

We re-plot Figures 2B and 2C to include all beta coefficients across our QAP models, irrespective of whether they were statistically significant or not at a p-value of 0.05 using a two-tailed t-test.

- Supplementary Figure 30 corresponds to Figure 2B.
- Supplementary Figure 31 corresponds to Figure 2C.

Supplementary Table of Fields Present Organized by Research Area

Supplementary Table 1.

Supplementary Tables Countries Present Organized by Core and Periphery

Supplementary Table 2.

Hierarchical Linear Models (HLMs)

We run two sets of hierarchical linear models (HLMs) set up in the same way, nesting countries in their respective fields. The purpose of these models is to see how much variance in our citational distortion and thus our text similarity measures are due to the result of the sheer volume of papers produced by authors from countries. Our focal independent variable is the number of papers that have at least one author from that country in $Field_t$. We also include two control variables: (1) the number of universities that a country has ranked are ranked in the top 50 of global universities on one of several university ranking schemes (e.g., CWUR-the Center for World University Ranking, the Times Higher Education, or US News and World Report) and (2) the percentage of a country's GDP allocated to R&D in each year. Supplementary Table 3 is an HLM that regresses countries' indegree in the citational well while Supplementary Table 4 is set up in the same way but regresses countries' indegree in the text similarity network. The data are from 1980 to 2012 in both HLM setups. The independent variables are z-score centered within fields. This can be interpreted as a one standard deviation increase in variable *x* (e.g., a one standard deviation increase in the number of papers with an author from country *i*, a one standard deviation increase in the percentage of R&D of GDP of country *i*, or a one standard deviation increase in the number of top 50 universities in country *i*) is associated with a standard deviation change in either the citational distortion centrality or the text similarity centrality. (Both sets of models in Supplementary Tables S3 and S4 are set up in the same way: Model 1 establishes the baseline variance without any independent variables, Models 2 through 4 introduce the three independent variables independently, and Model 5 includes all three variables. Across both sets of models, the variance is quite low, 0.07 in Model 5 for the citational distortion HLM (Supplementary Table 3) and 0.13 in Model 5 for the text similarity HLM (Supplementary Table 4). The volume of papers does not explain the variance in the trends we show in the main paper.

Supplementary Table 3.

Supplementary Table 4.

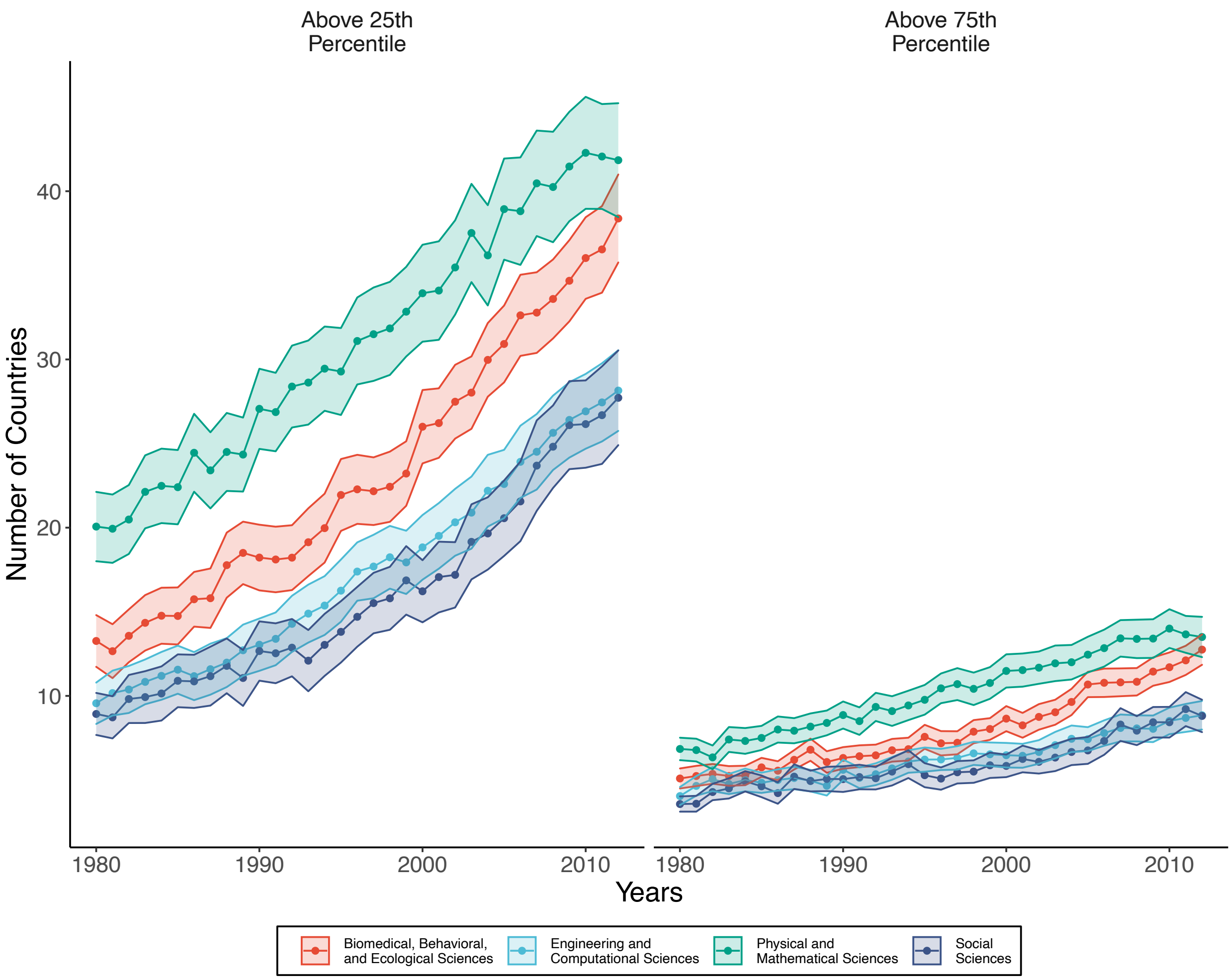

Supplementary Figure 1. Number of countries engaged in science by topic coherence. We plot the number of countries present in fields in each year from 1980 to 2012, with trend lines for each field type. The shading around the trends denotes the standard errors to these averages.

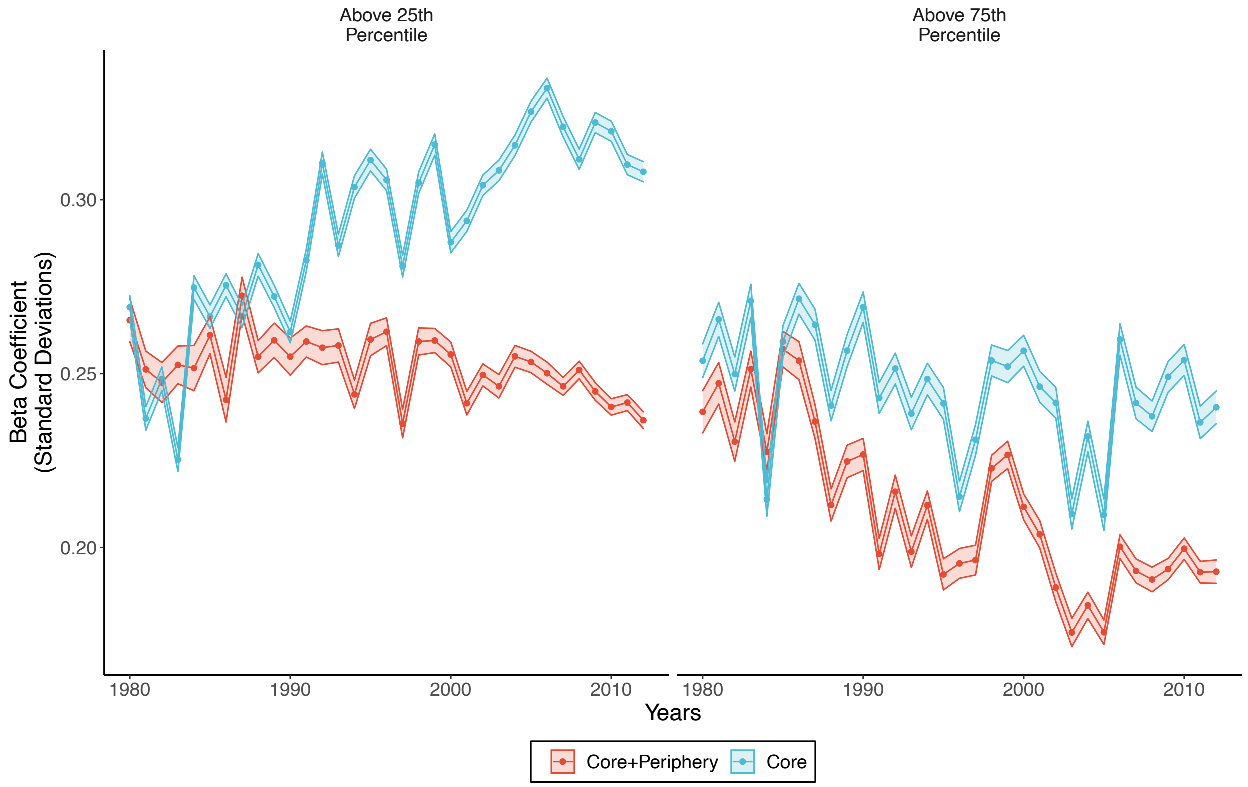

Supplementary Figure 2. Relationship between citations and text over time by topic coherence. We plot the average of statistically-significant beta coefficients from each field's yearly QAP model for citations plotted over time from 1980 to 2012. We do this where we include nation-labels whose topic coherence scores are above the 25th and the 75th percentile. The shading around the trends denotes the grand standard errors across beta-coefficients.

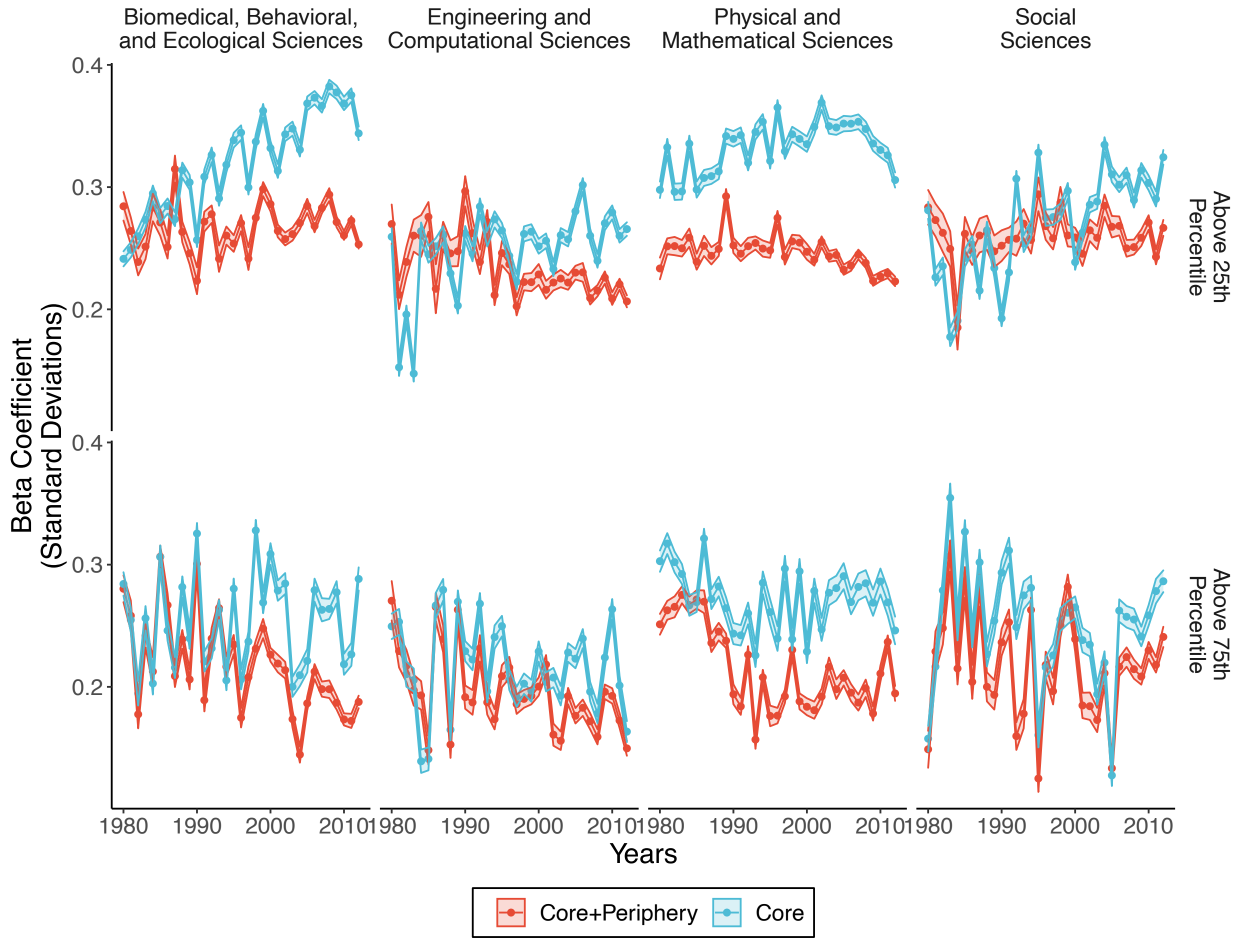

Supplementary Figure 3. Relationship between citations and text over time by topic coherence and research area. We plot the trends in Supplementary Figure 2 but parsed by the type of field. We do this where we include nation-labels whose topic coherence scores are above the 25th and the 75th percentile. The shading around the trends denotes the grand standard errors across beta-coefficients.

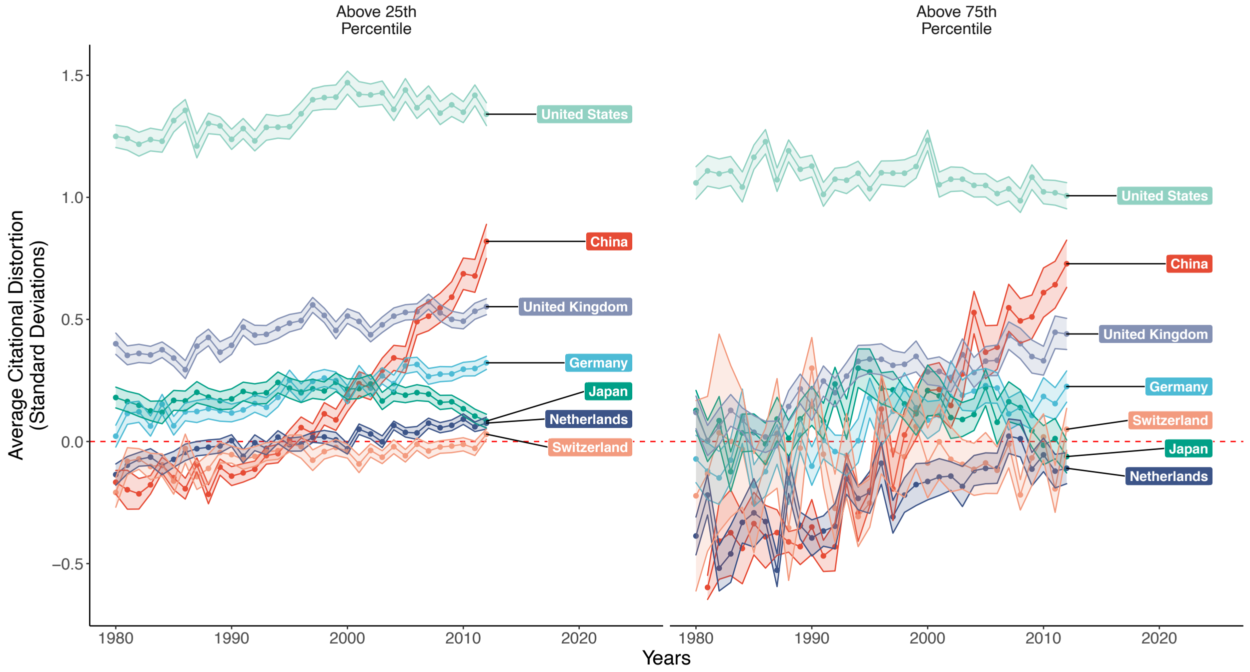

Supplementary Figure 4. Average citational distortion for countries over time by topic coherence. We plot the average national citational distortion in $\boldsymbol{L_{distortion}}$ across fields and plotted over time. We do this where we include nation-labels whose topic coherence scores are above the 25th percentile and the 75th percentile. The shading around the trends denotes the standard errors to these averages.

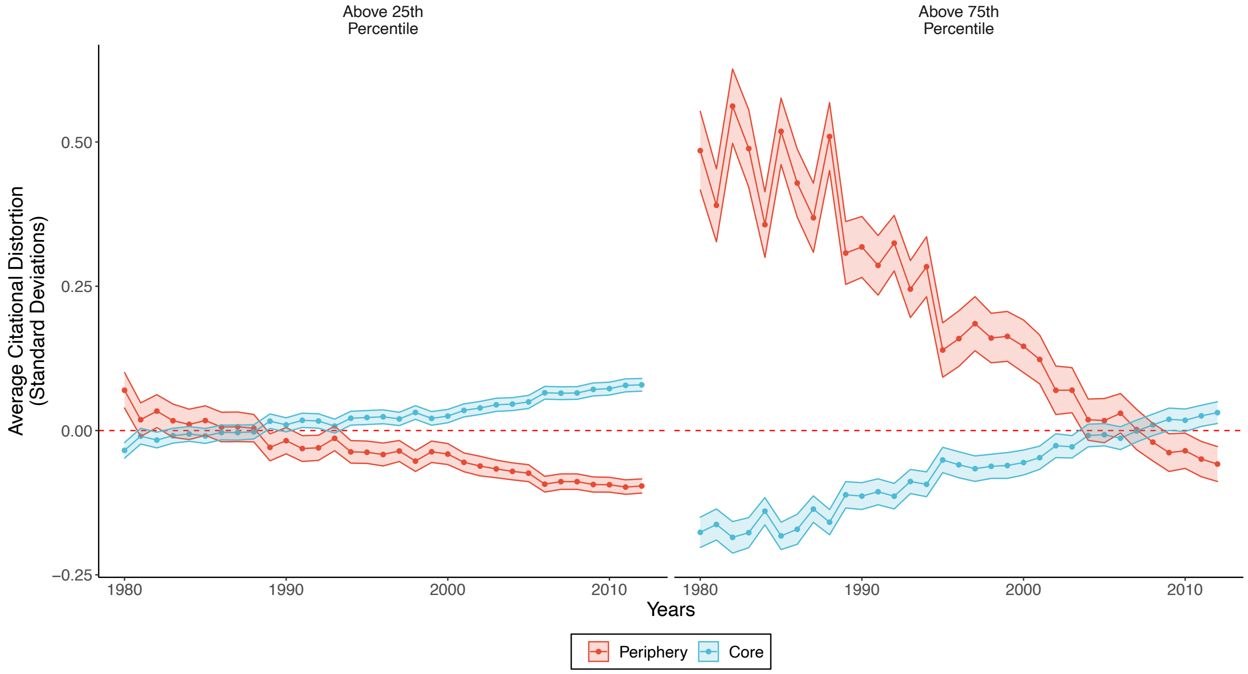

Supplementary Figure 5. Average citational distortion for core and periphery countries over time by topic coherence. We plot the average national citational distortion in $\boldsymbol{L_{distortion}}$ between core and periphery countries across fields and plotted over time. We do this where we include nation-labels whose topic coherence scores are above the 25th percentile and the 75th percentile. The shading around the trends denotes the standard errors to these averages.

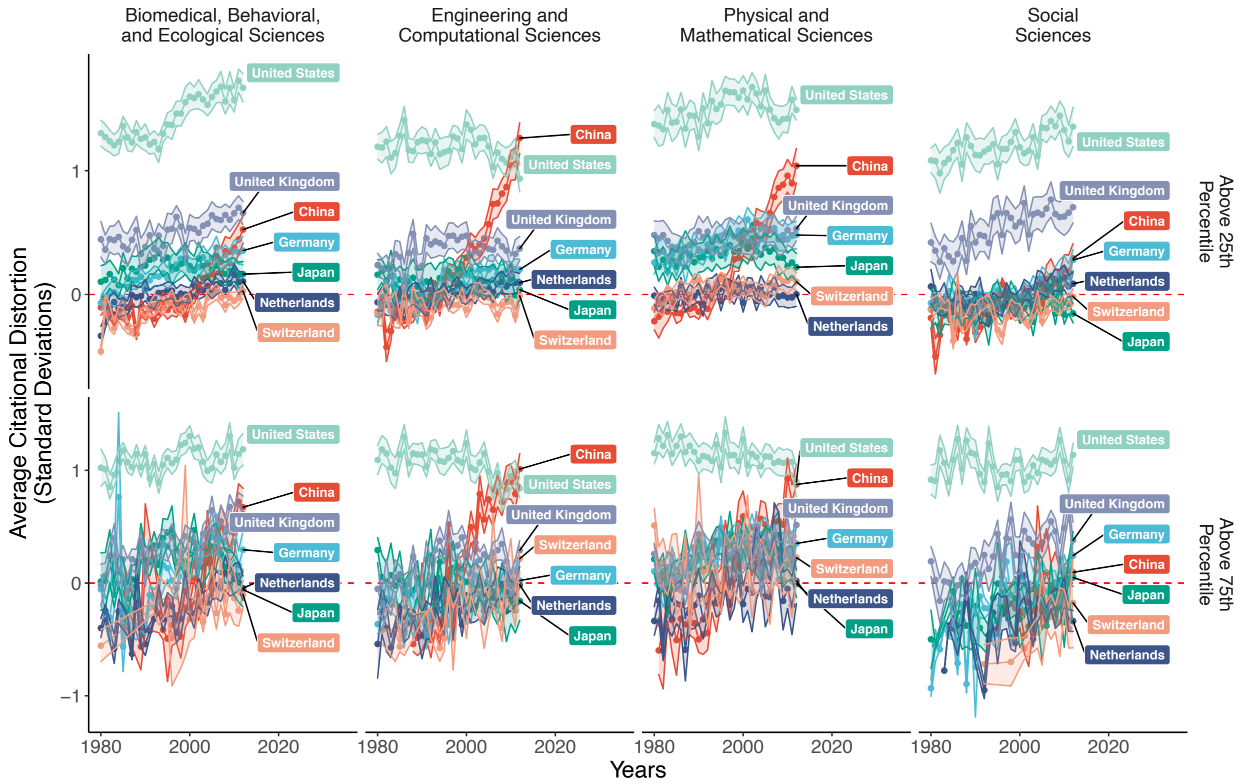

Supplementary Figure 6. Average citational distortion for countries over time by topic cohesion and by research area. Plots the trends in Supplementary Figure 4 but parsed by the type of field. We do this where we include nation-labels whose topic coherence scores are above the 25th percentile and the 75th percentile. The shading around the trends denotes the standard errors to these averages.

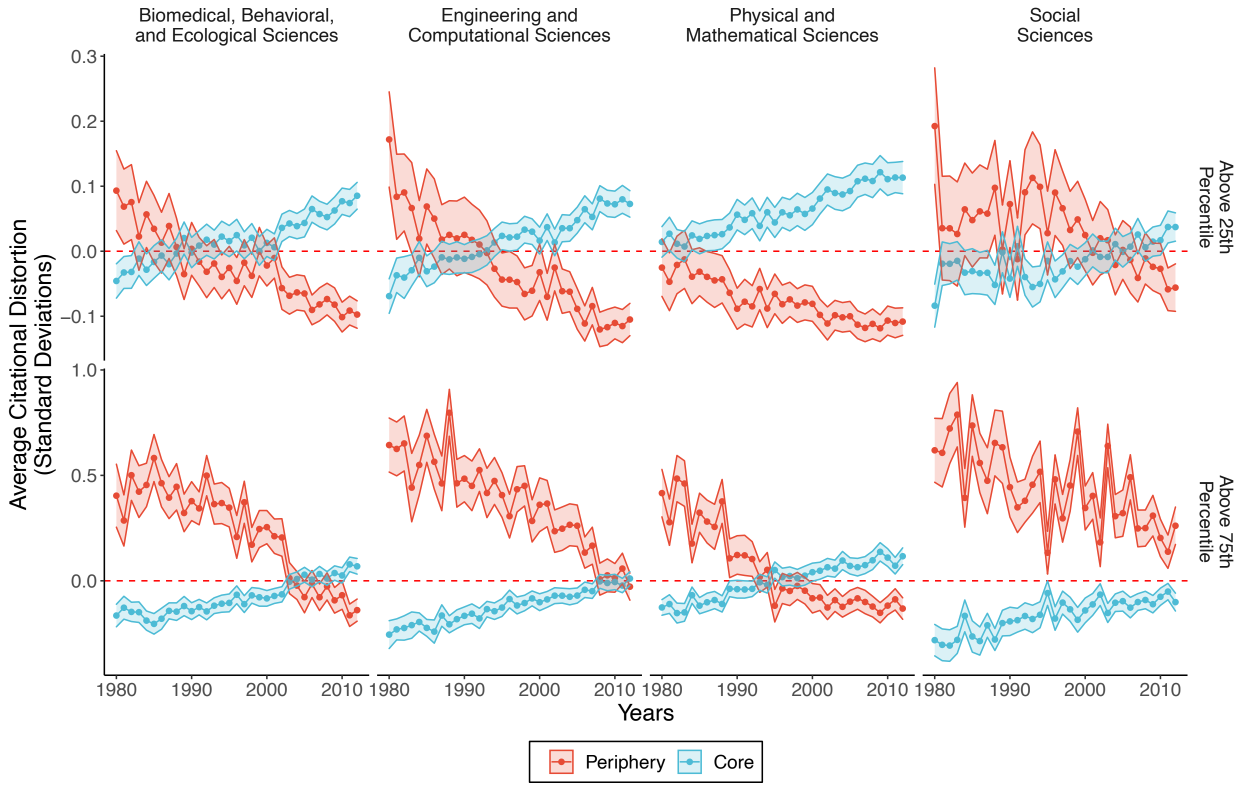

Supplementary Figure 7. Average citational distortion for core and periphery countries over time by topic cohesion and by research area. Plots the trends in Supplementary Figure 5 but parsed by the type of field. We do this where we include nation-labels whose topic coherence scores are above the 25th percentile and the 75th percentile. The shading around the trends denotes the standard errors to these averages.

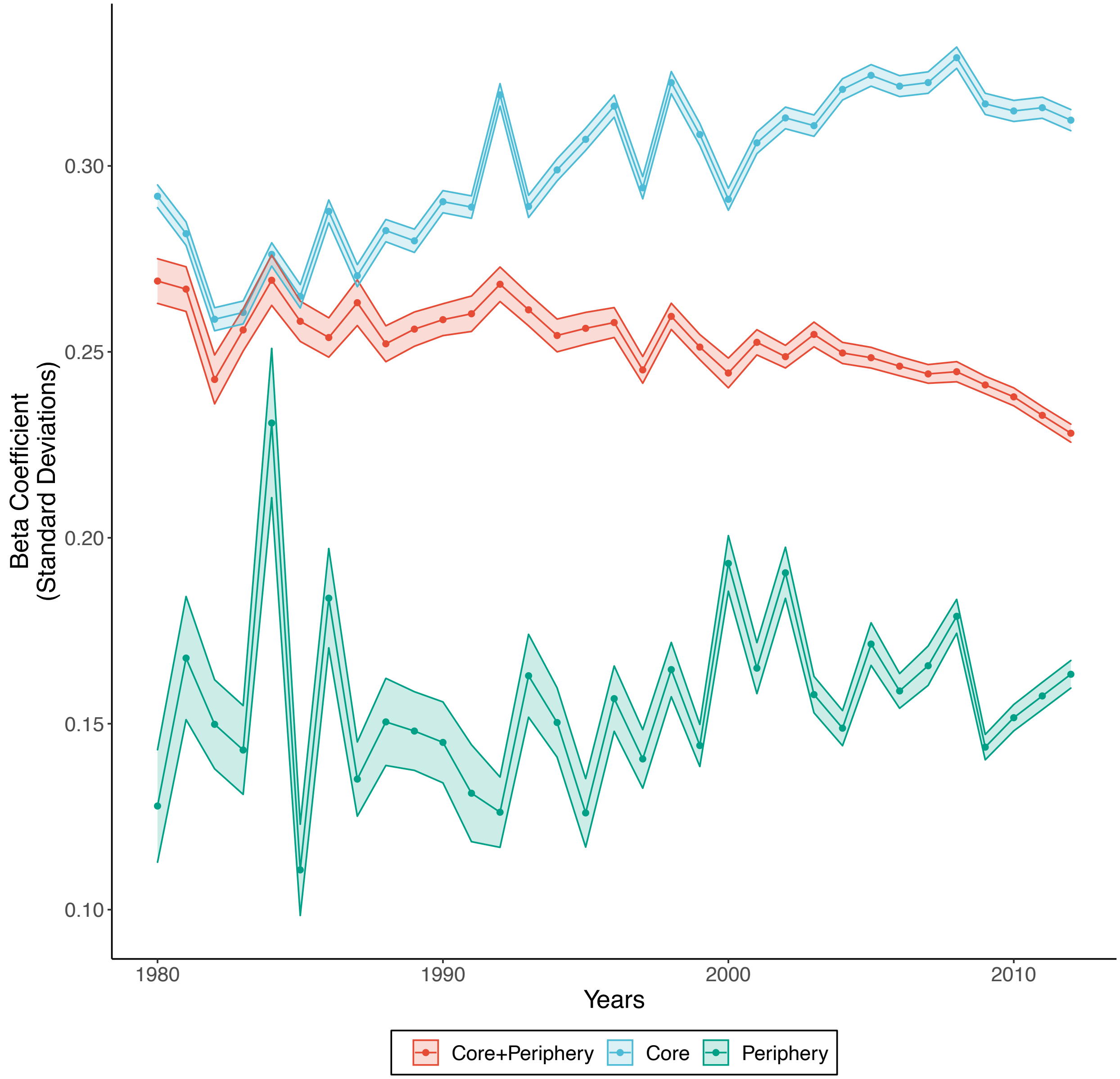

Supplementary Figure 8. Relationship between citations and text over time for all countries, core, and only periphery. We plot the average of statistically-significant beta coefficients from each field's yearly QAP model for citations plotted over time from 1980 to 2012. We include a third trend line for countries that are not at the "core" of global science, labeled as "periphery." The shading around the trends denotes the grand standard errors across beta-coefficients.

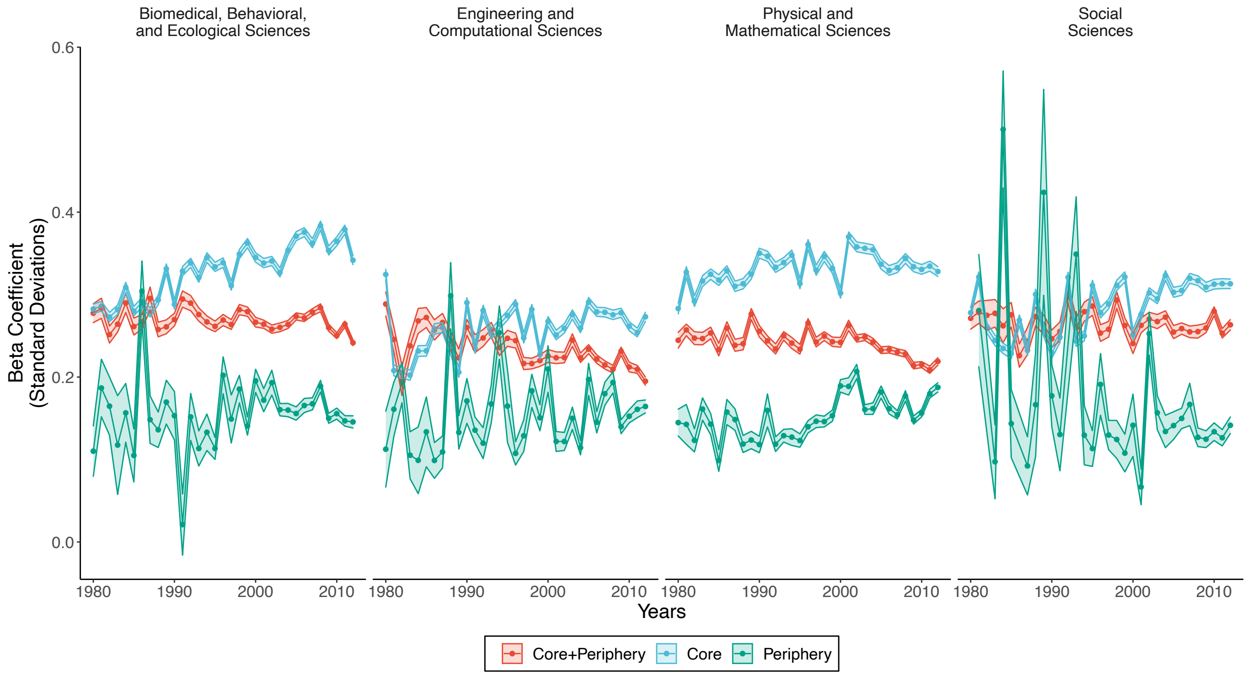

Supplementary Figure 9. Relationship between citations and text over time for all countries, core, and only periphery by research area. Plots the trends in Supplementary Figure 11 but parsed by the type of field. We plot the average of statistically-significant beta coefficients from each field's yearly QAP model for citations plotted over time from 1980 to 2012. We include a third trend line for countries that are not at the "core" of global science, labeled as "periphery." The shading around the trends denotes the grand standard errors across beta-coefficients.

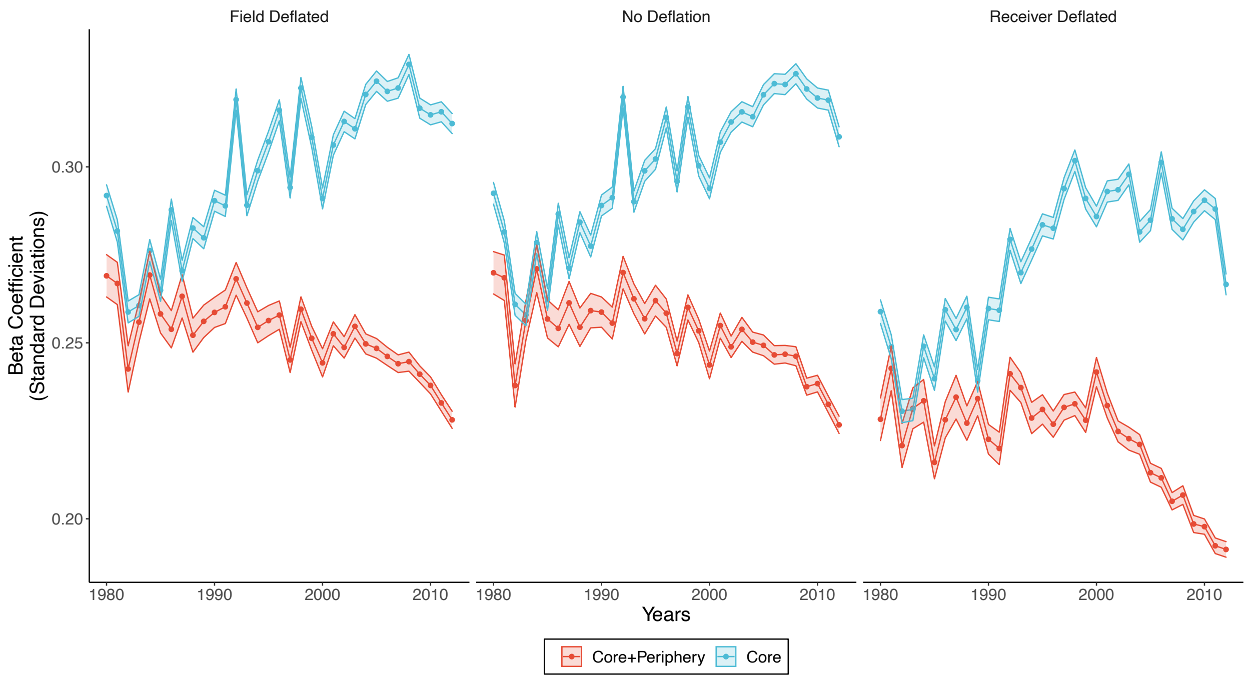

Supplementary Figure 10. Relationship between citations and text comparing citation deflation over time. We plot the average of statistically-significant beta coefficients from each field's yearly QAP model for citations plotted over time from 1980 to 2012. We include two panes: one where the citation networks are "deflated" following Petersen, et al.[12] and one where the citation networks were not deflated. The shading around the trends denotes the grand standard errors across beta-coefficients.

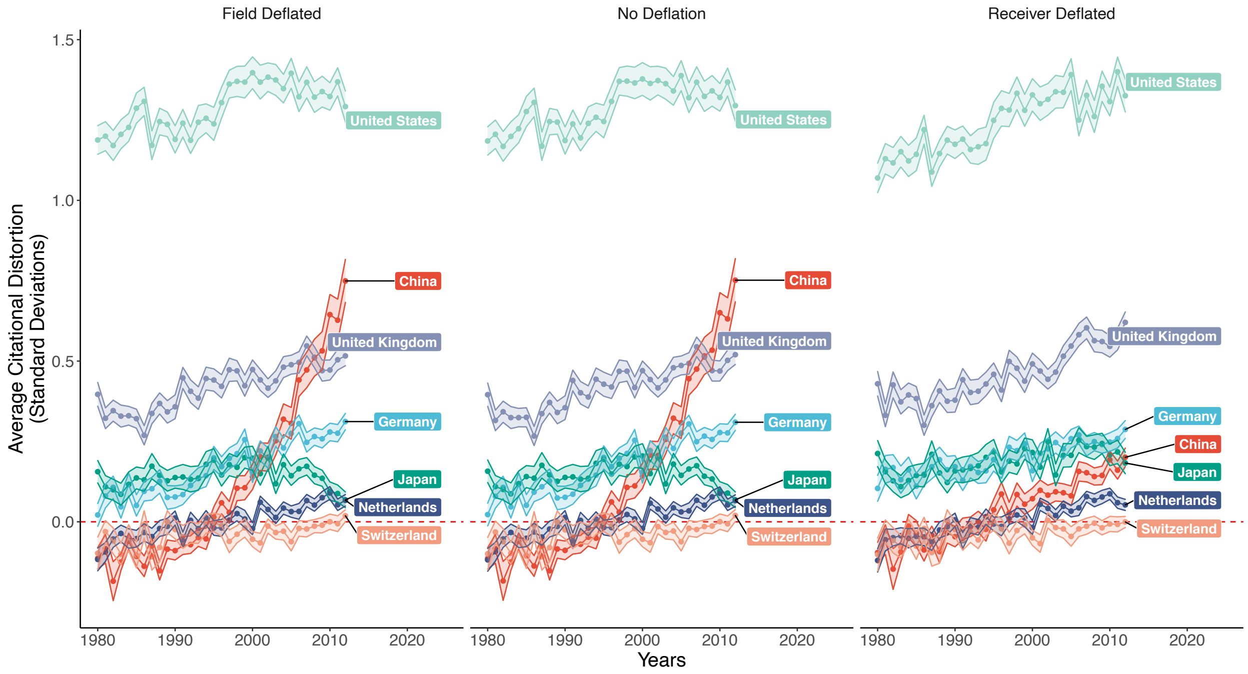

Supplementary Figure 12. Average citational distortion for countries comparing citation deflation over time. We plot the average national citational distortion in $\boldsymbol{L_{distortion}}$ across fields and plotted over time. We include three panes: one where the citation networks are "deflated" following Petersen, et al.[12], one where the centralities are "deflated," and one where the citation networks were not deflated. The shading around the trends denotes the standard errors to these averages.

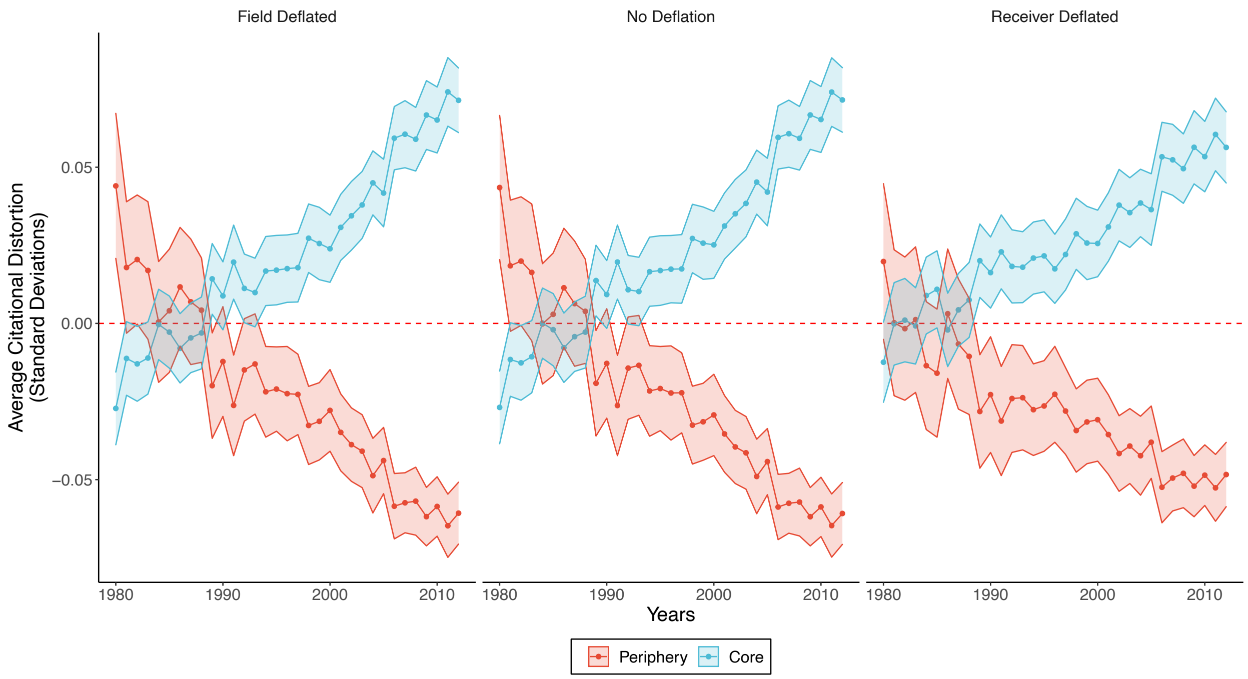

Supplementary Figure 13. Average citational distortion for core and periphery countries comparing citation deflation over time. We plot the average national citational distortion in $L_{distortion}$ between core and periphery countries across fields and plotted over time. We include three panes: one where the citation networks are "deflated" following Petersen, et al.[12], one where the centralities are "deflated," and one where the citation networks were not deflated. The shading around the trends denotes the standard errors to these averages.

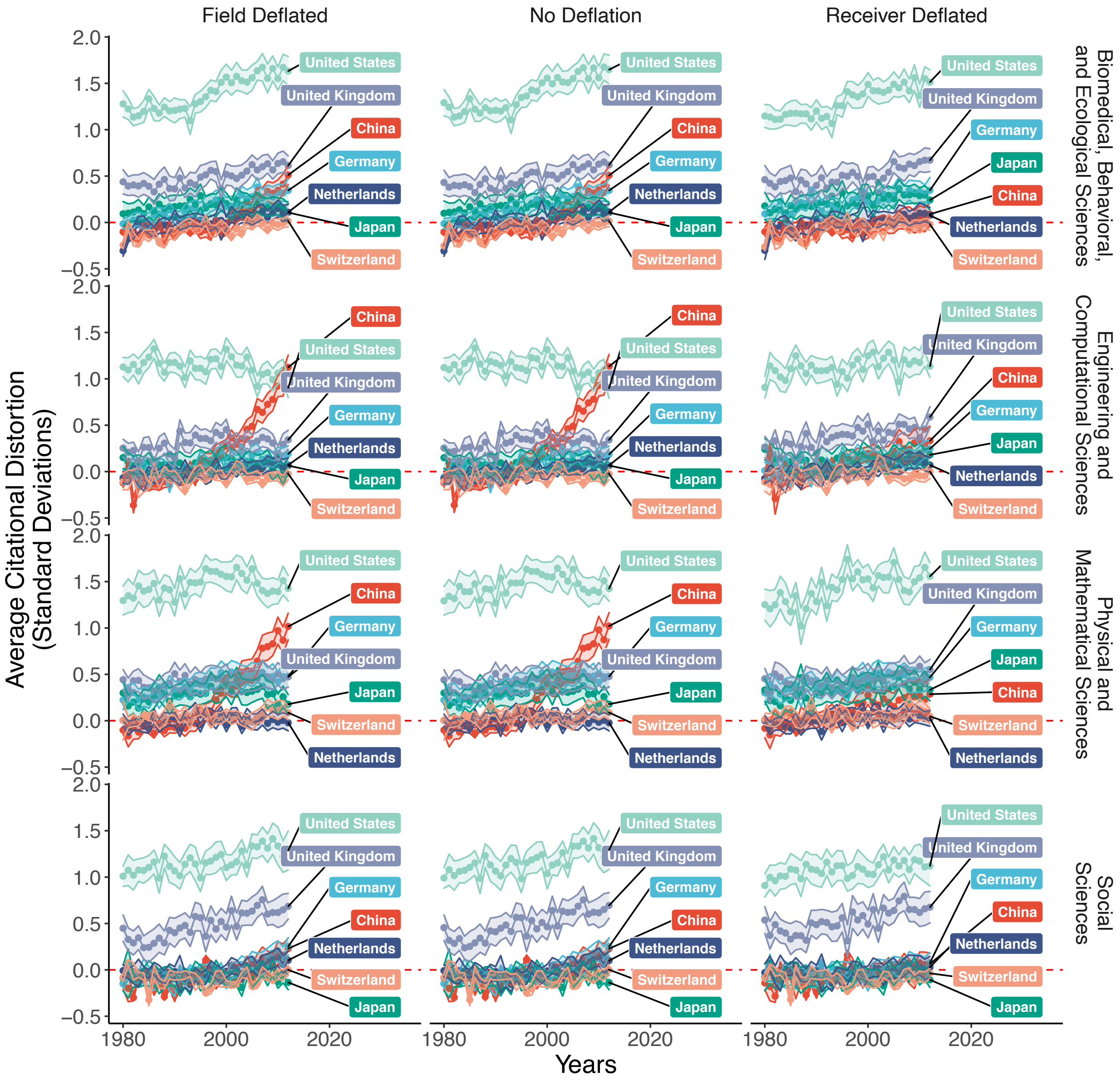

Supplementary Figure 14. Average citational distortion for countries comparing citation deflation over time by research area. Plots the trends in Supplementary Figure 12 but parsed by the type of field. We include three panes: one where the citation networks are “deflated” following Petersen, et al.[12], one where the centralities are “deflated,” and one where the citation networks were not deflated. The shading around the trends denotes the standard errors to these averages.

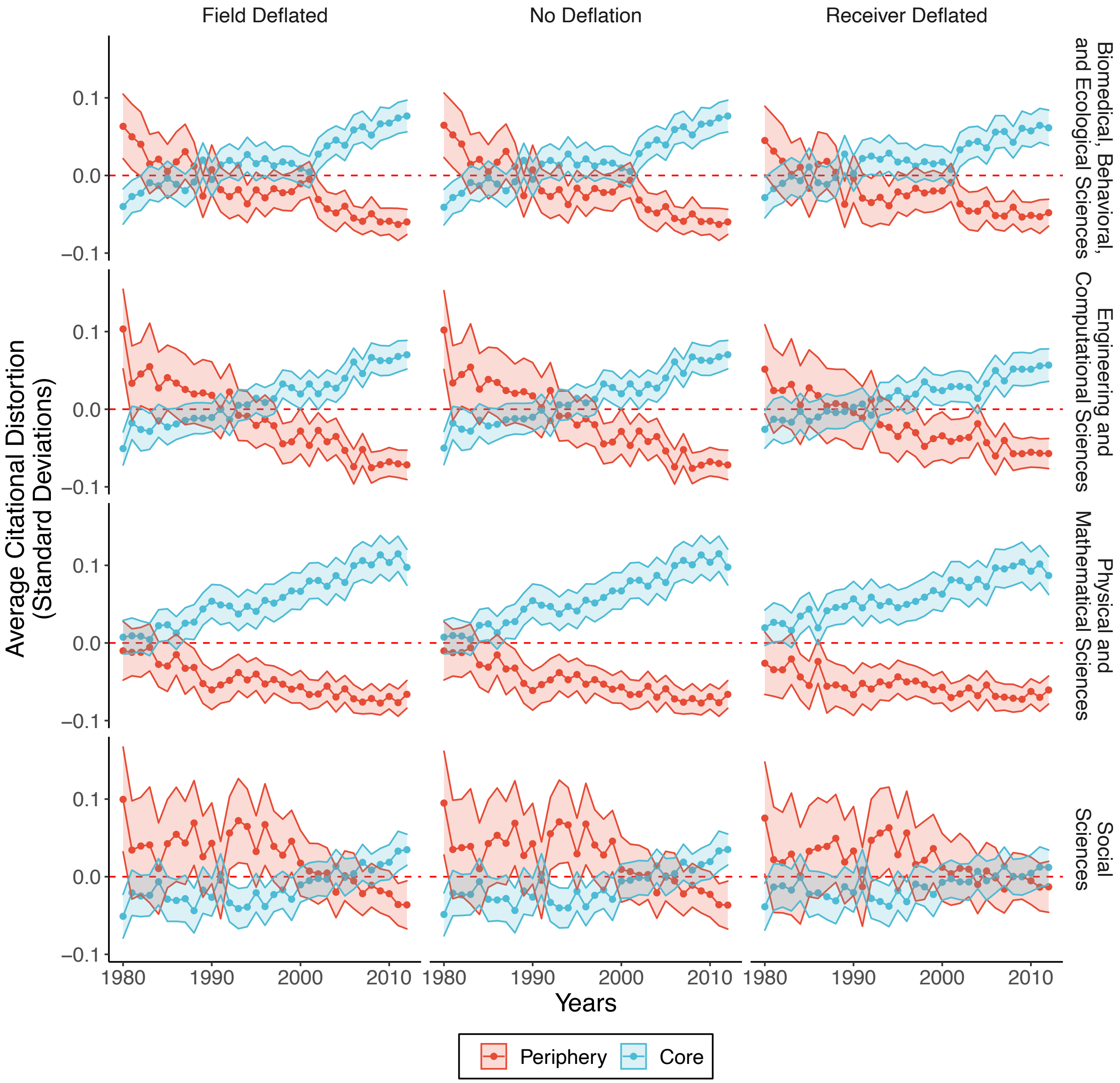

Supplementary Figure 15. Average citational distortion for core and periphery countries comparing citation deflation over time by research area. Plots the trends in Supplementary Figure 13 but parsed by the type of field. We include three panes: one where the citation networks are "deflated" following Petersen, et al.[12], one where the centralities are "deflated," and one where the citation networks were not deflated. The shading around the trends denotes the standard errors to these averages.

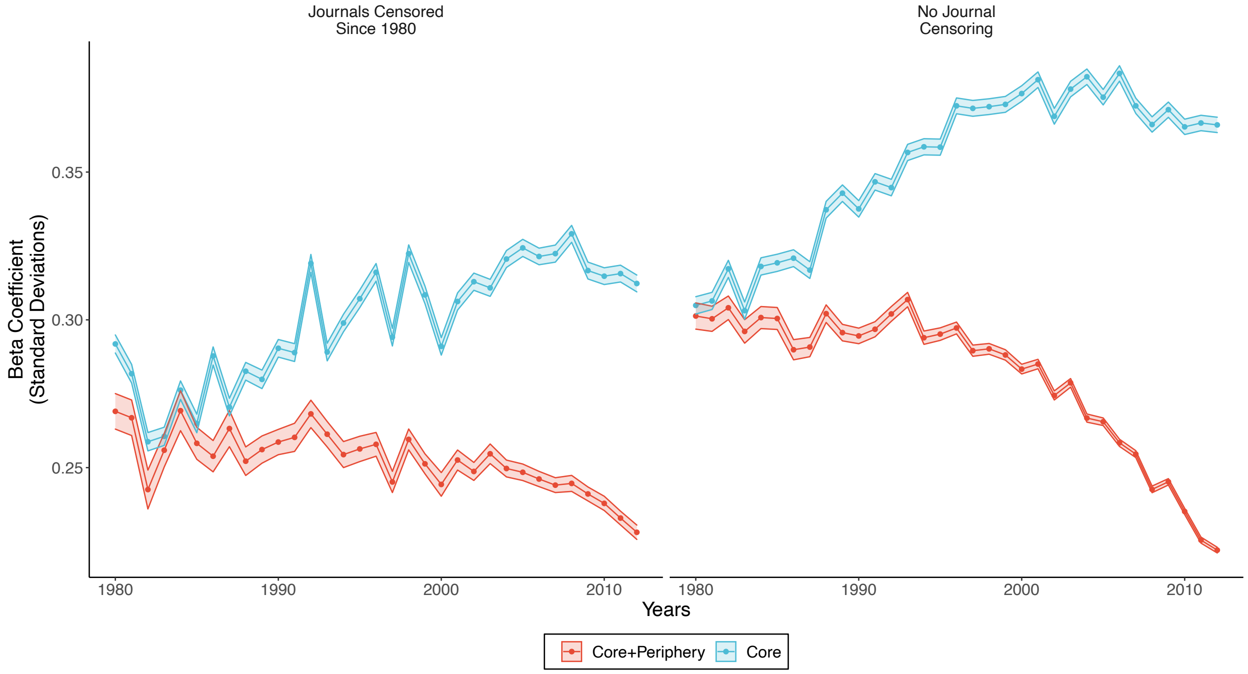

Supplementary Figure 16. Relationship between citations and text comparing journal censoring over time. We plot the average of statistically-significant beta coefficients from each field's yearly QAP model for citations plotted over time from 1980 to 2012. We include two panes: one where the analyses were built using all journals present in the data and another where we censor for journals that have existed since the year 1980. The shading around the trends denotes the grand standard errors across beta-coefficients.

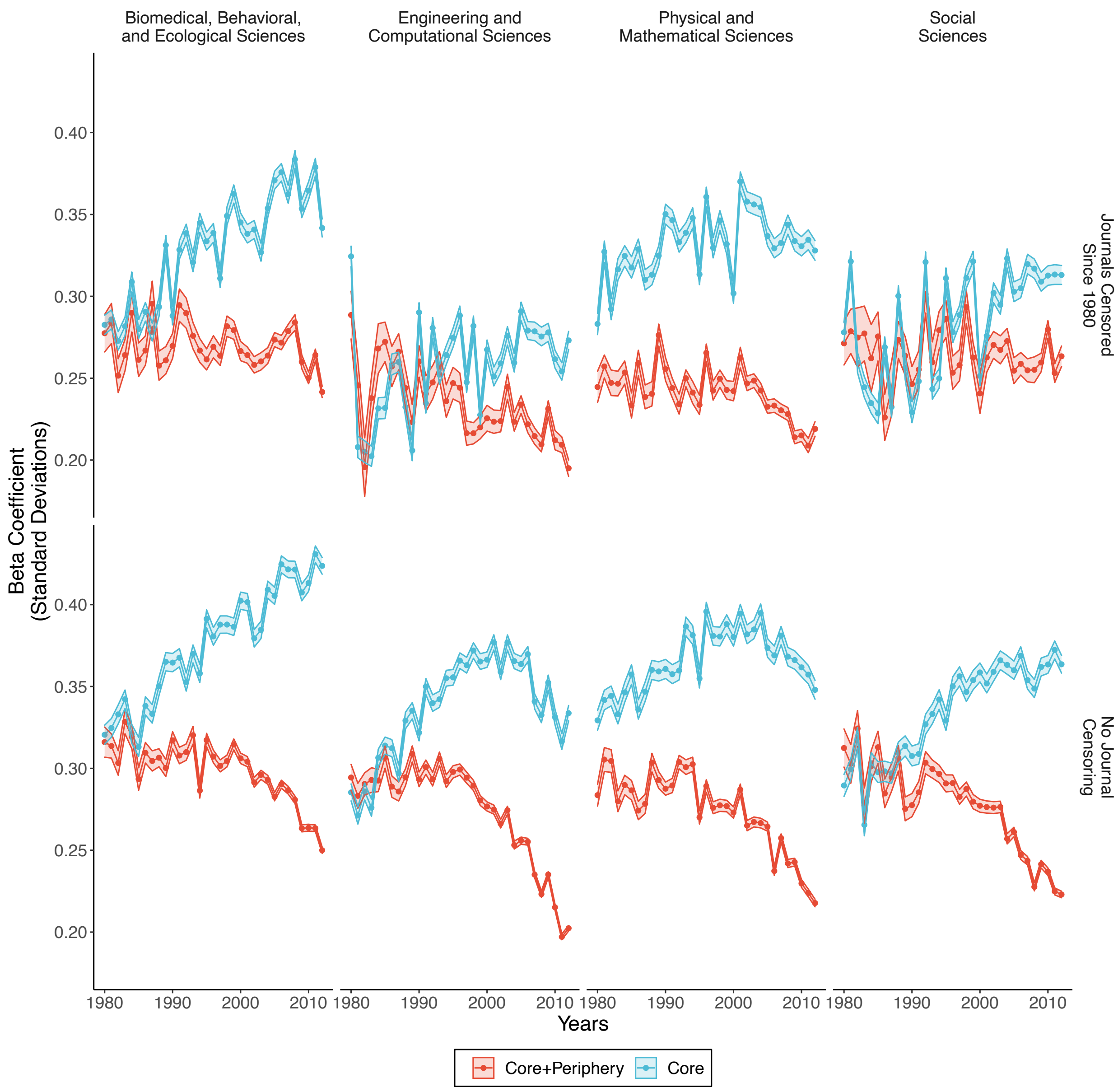

Supplementary Figure 17. Relationship between citations and text comparing journal censoring over time by research area. Plots the trends in Supplementary Figure 19 but parsed by the type of field. We include two panes: one where the analyses were built using all journals present in the data and another where we censor for journals that have existed since the year 1980. The shading around the trends denotes the grand standard errors across beta-coefficients.

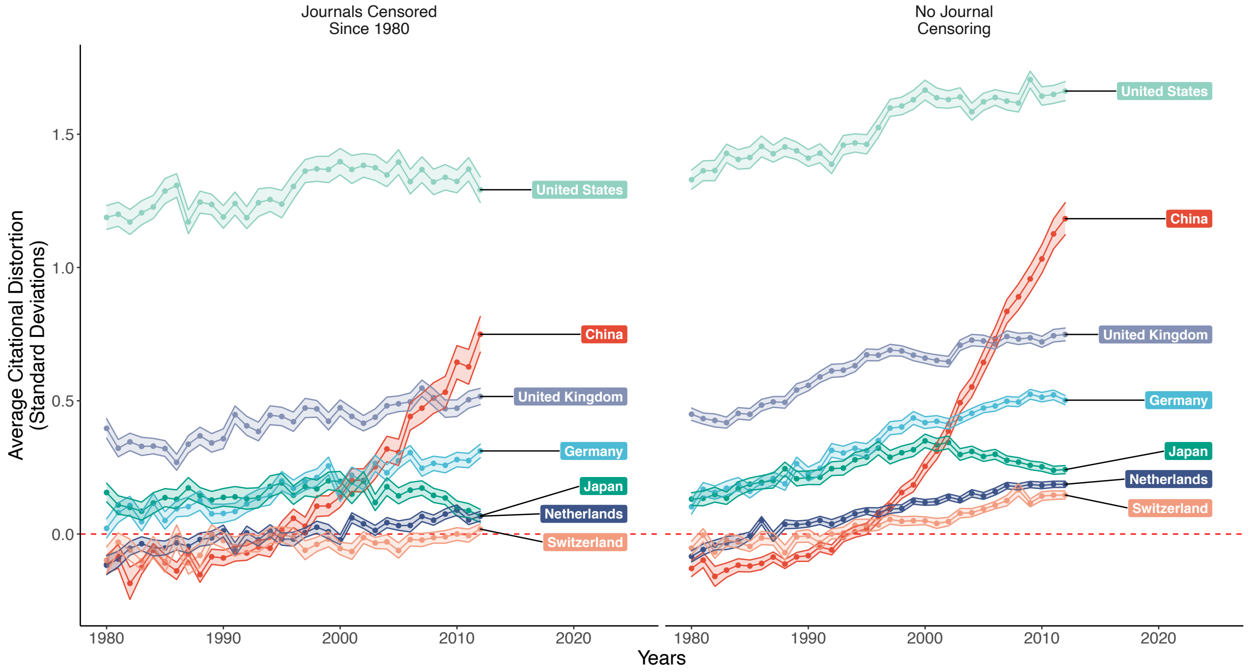

Supplementary Figure 18. Average citational distortion for countries comparing journal censoring over time. We plot the average national citational distortion in $\boldsymbol{L_{distortion}}$ across fields and plotted over time. We include two panes: one where the analyses were built using all journals present in the data and another where we censor for journals that have existed since the year 1980. The shading around the trends denotes the standard errors to these averages.

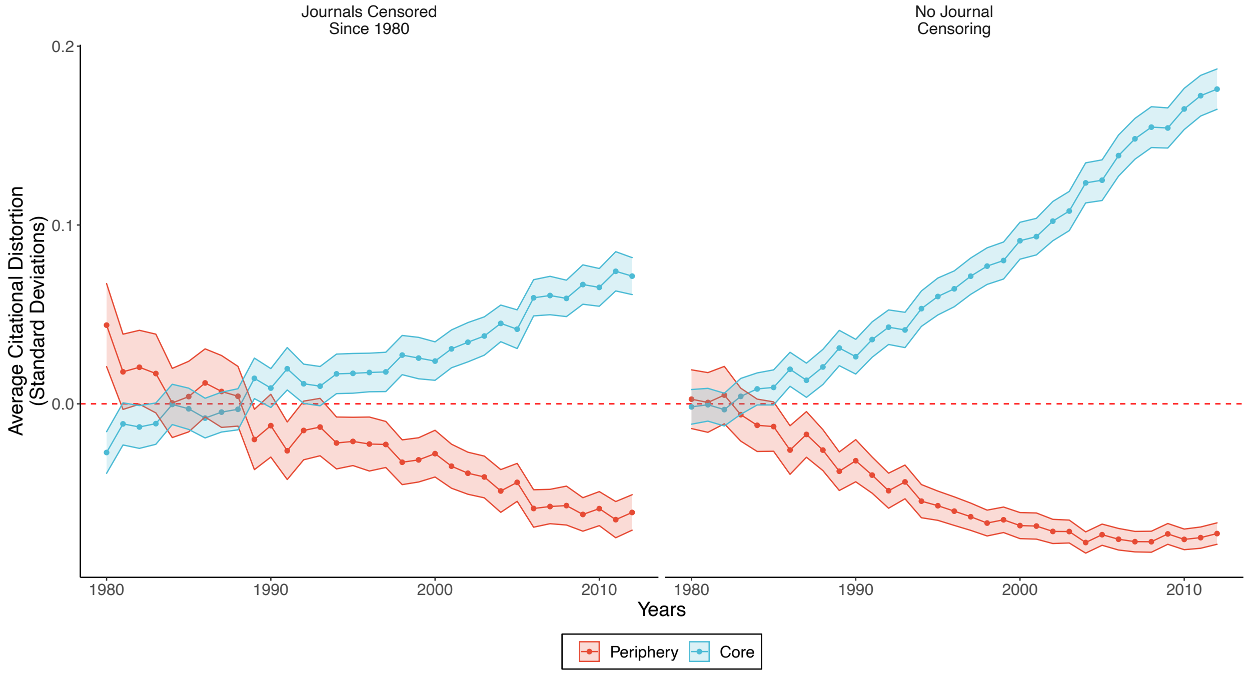

Supplementary Figure 19. Average citational distortion for core and periphery countries comparing journal censoring over time. We plot the average national citational distortion in $L_{distortion}$ between core and periphery countries across fields and plotted over time. We include two panes: one where the analyses were built using all journals present in the data and another where we censor for journals that have existed since the year 1980. The shading around the trends denotes the standard errors to these averages.

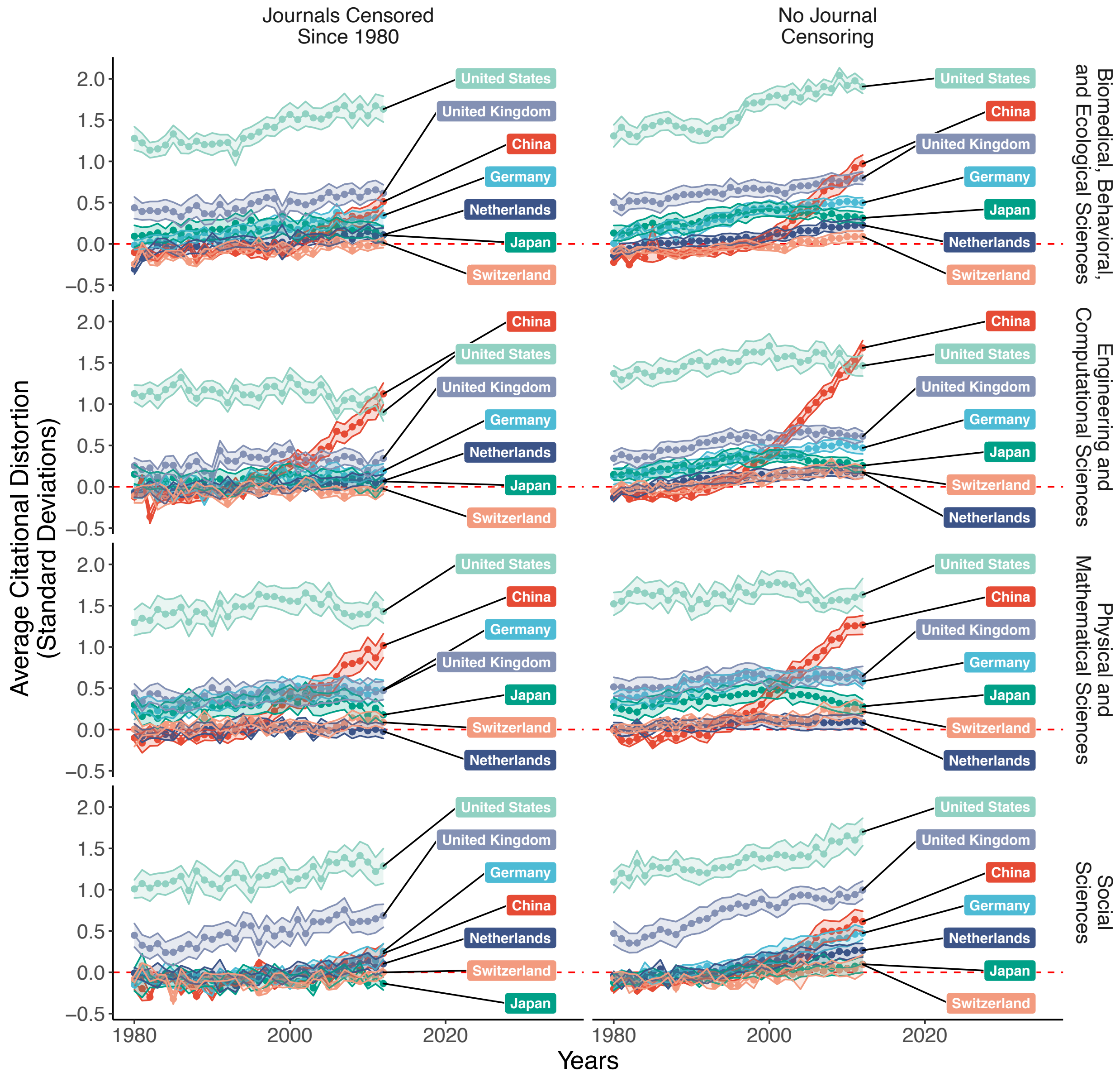

Supplementary Figure 20. Average citational distortion for countries comparing journal censoring over time by research area. Plots the trends in Supplementary Figure 18 but parsed by the type of field. We include two panes: one where the analyses were built using all journals present in the data and another where we censor for journals that have existed since the year 1980. The shading around the trends denotes the standard errors to these averages.

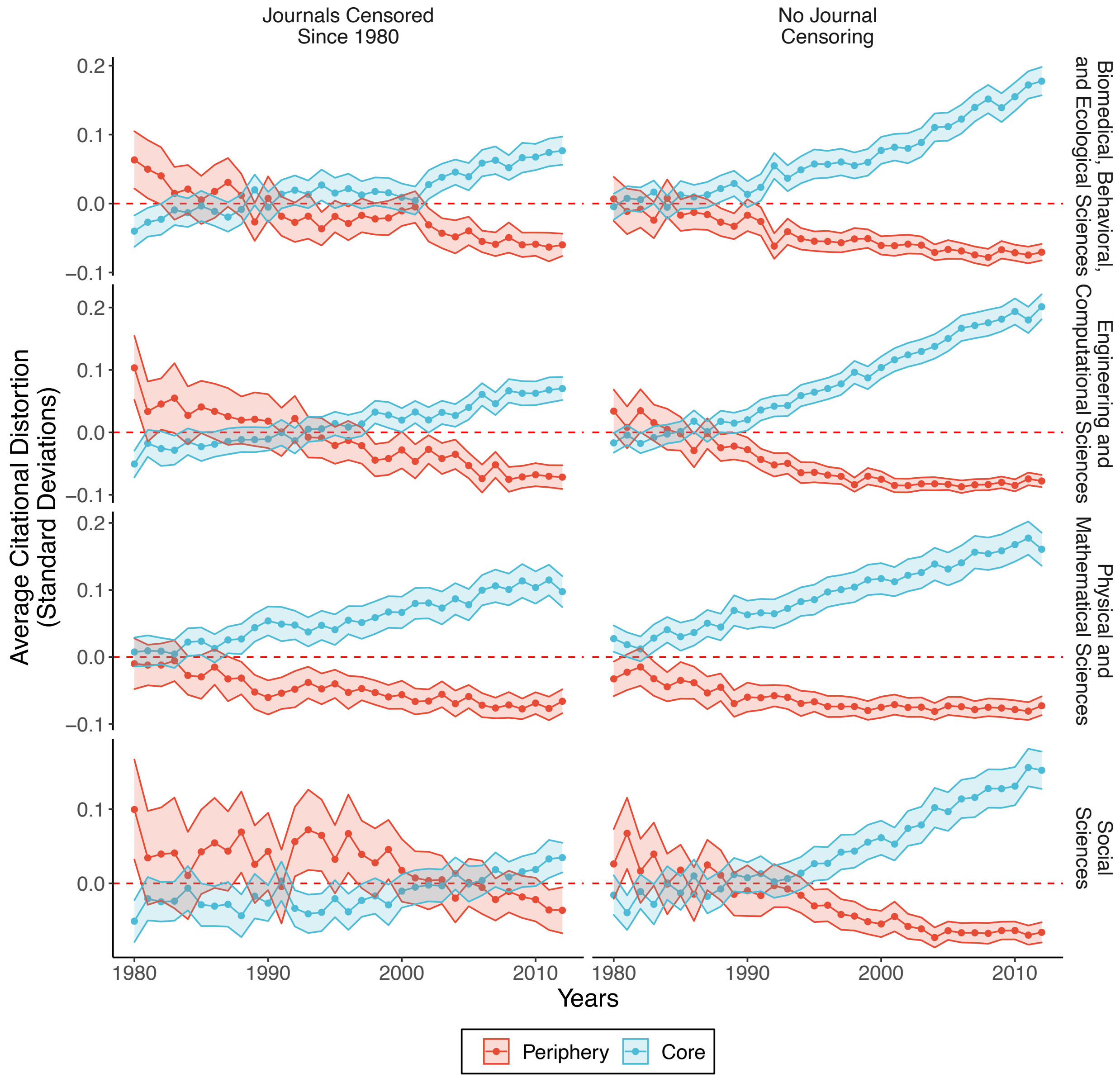

Supplementary Figure 21. Average citational distortion for core and periphery countries comparing journal censoring over time by research area. Plots the trends in Supplementary Figure 19 but parsed by the type of field. We include two panes: one where the analyses were built using all journals present in the data and another where we censor for journals that have existed since the year 1980. The shading around the trends denotes the standard errors to these averages.

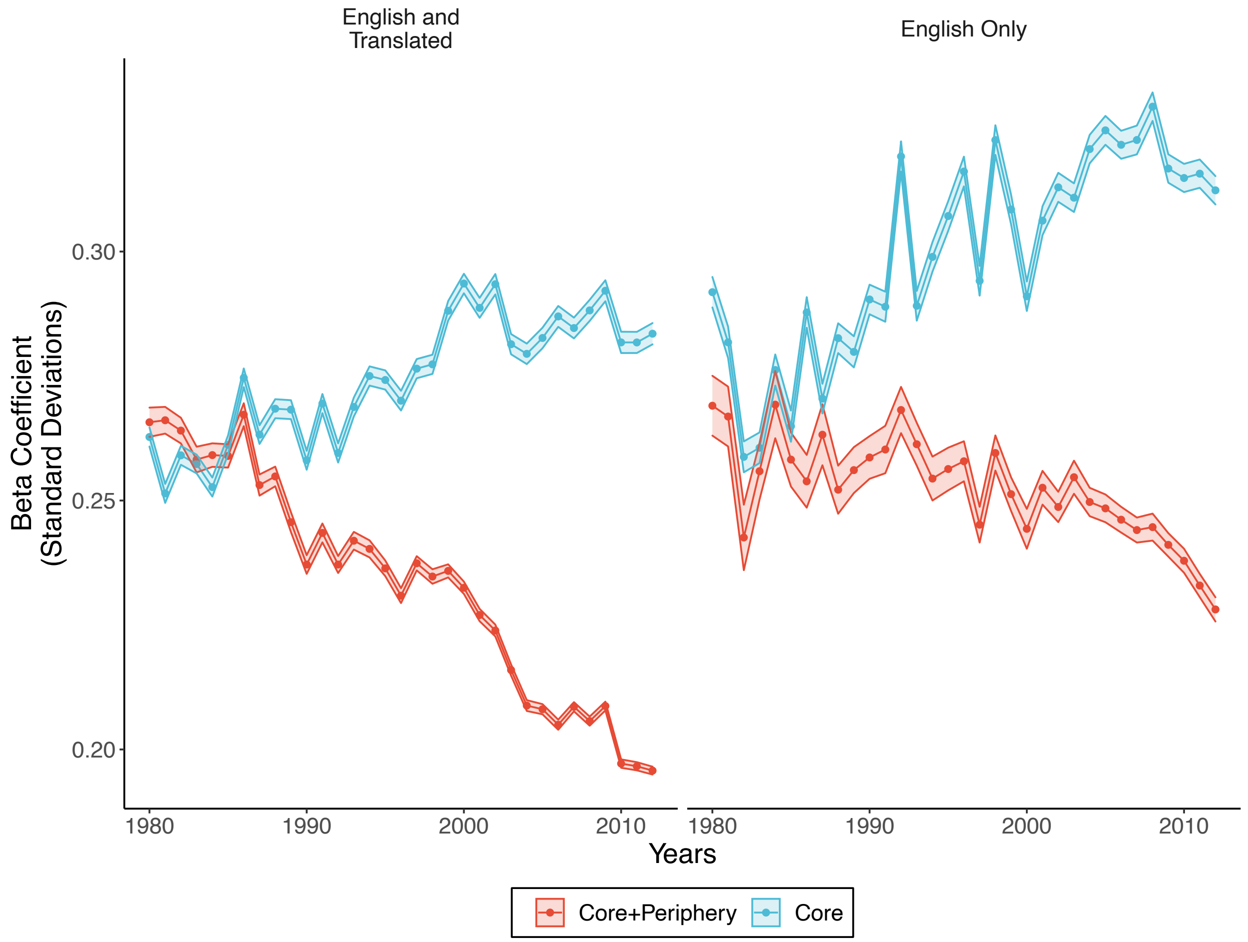

Supplementary Figure 22. Relationship between citations and text comparing English censored abstracts over time. We plot the average of statistically-significant beta coefficients from each field's yearly QAP model for citations plotted over time from 1980 to 2012. We include two panes: one where the networks were built from English and translated abstracts and one where the networks used English abstracts only. The shading around the trends denotes the grand standard errors across beta-coefficients.

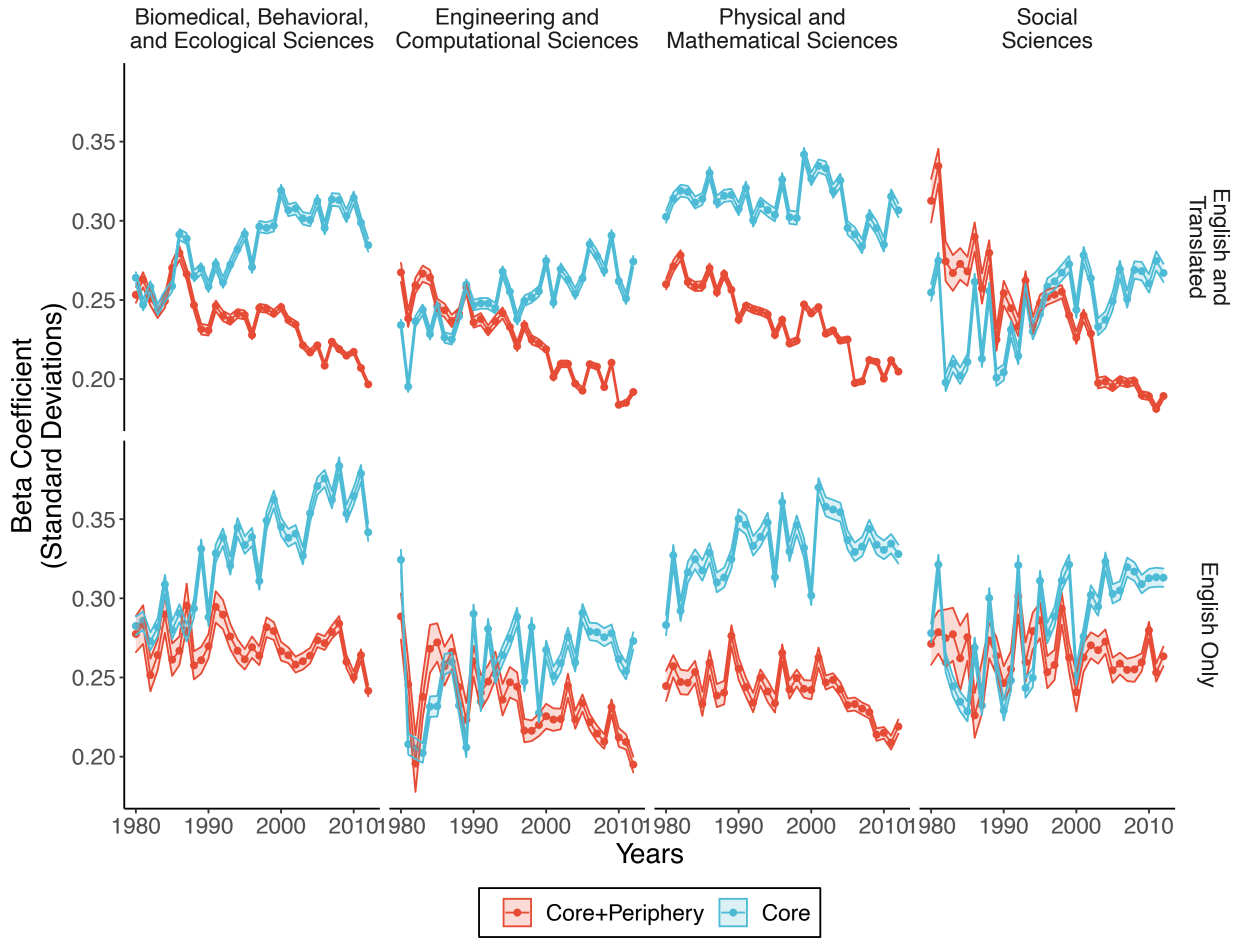

Supplementary Figure 23. Relationship between citations and text comparing English censored abstracts over time by research area. Plots the trends in Supplementary Figure 22 but parsed by the type of field. We include two panes: one where the networks were built from English and translated abstracts and one where the networks used English abstracts only. The shading around the trends denotes the grand standard errors across beta-coefficients.

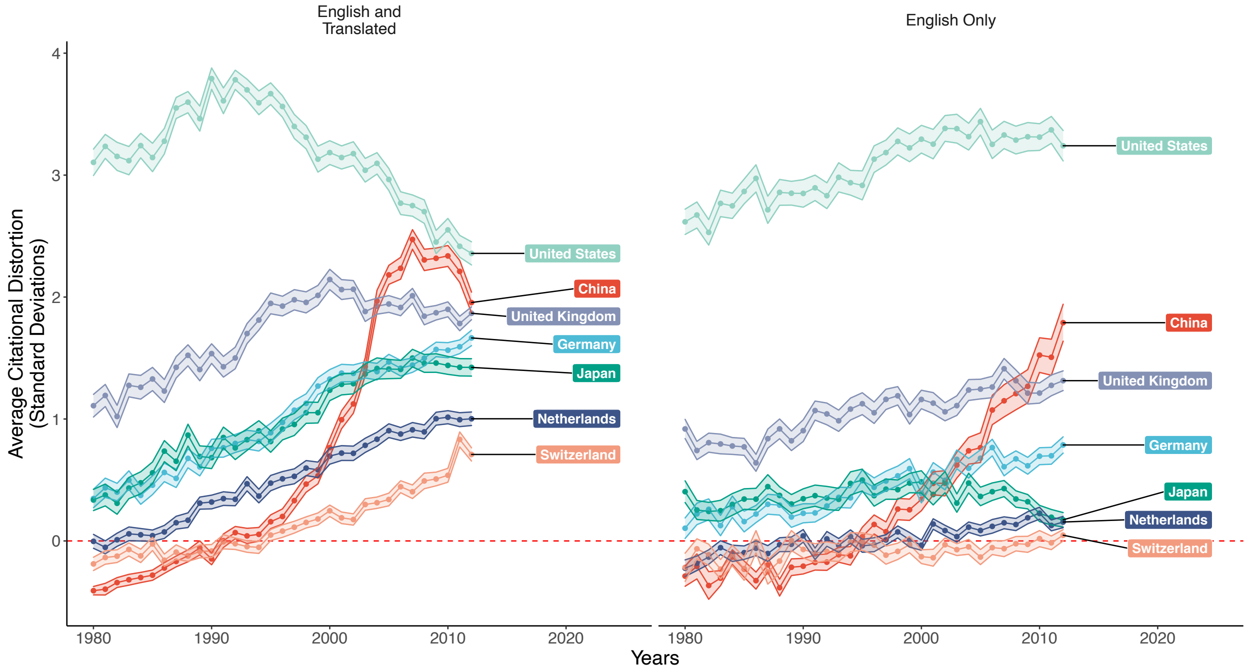

Supplementary Figure 24. Average citational distortion for countries comparing English censored abstracts over time. We plot the average national citational distortion in $L_{distortion}$ across fields and plotted over time. We include two panes: one where networks were built from English and translated abstracts and one where the networks used English abstracts only. The shading around the trends denotes the standard errors to these averages.

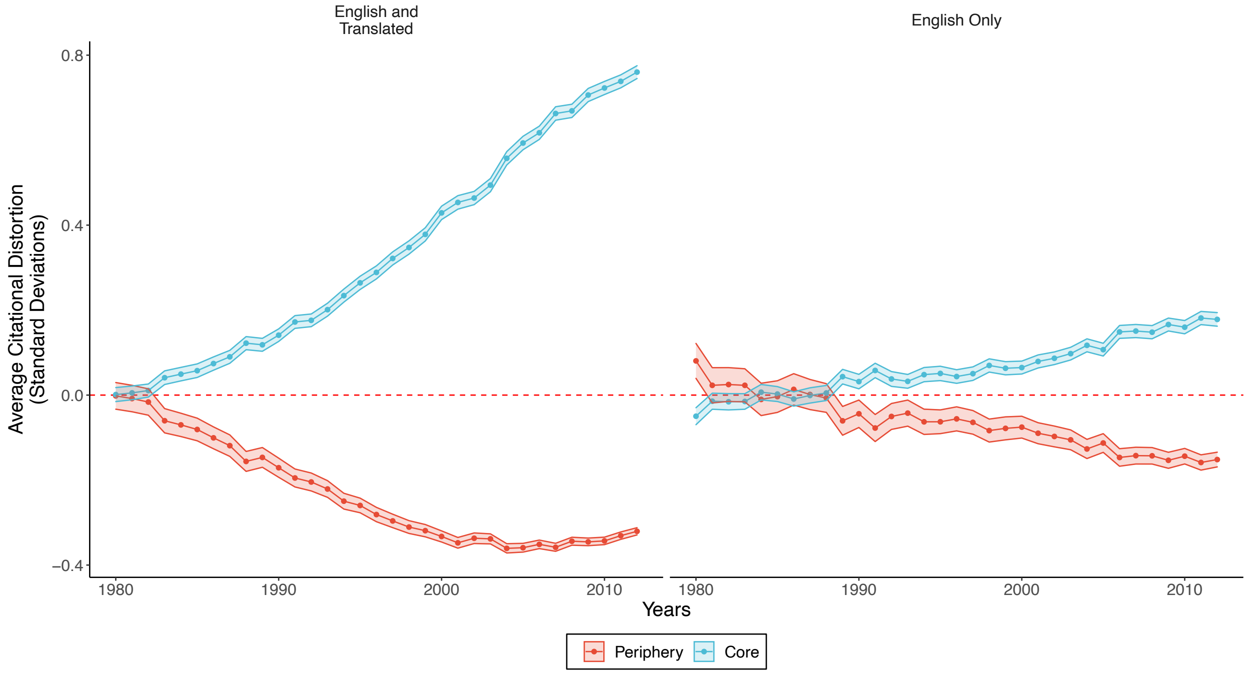

Supplementary Figure 25. Average citational distortion for core and periphery countries comparing English censored abstracts over time. We plot the average national citational distortion in $\boldsymbol{L_{distortion}}$ across fields and plotted over time. We include two panes: one where networks were built from English and translated abstracts and one where the networks used English abstracts only. The shading around the trends denotes the standard errors to these averages.

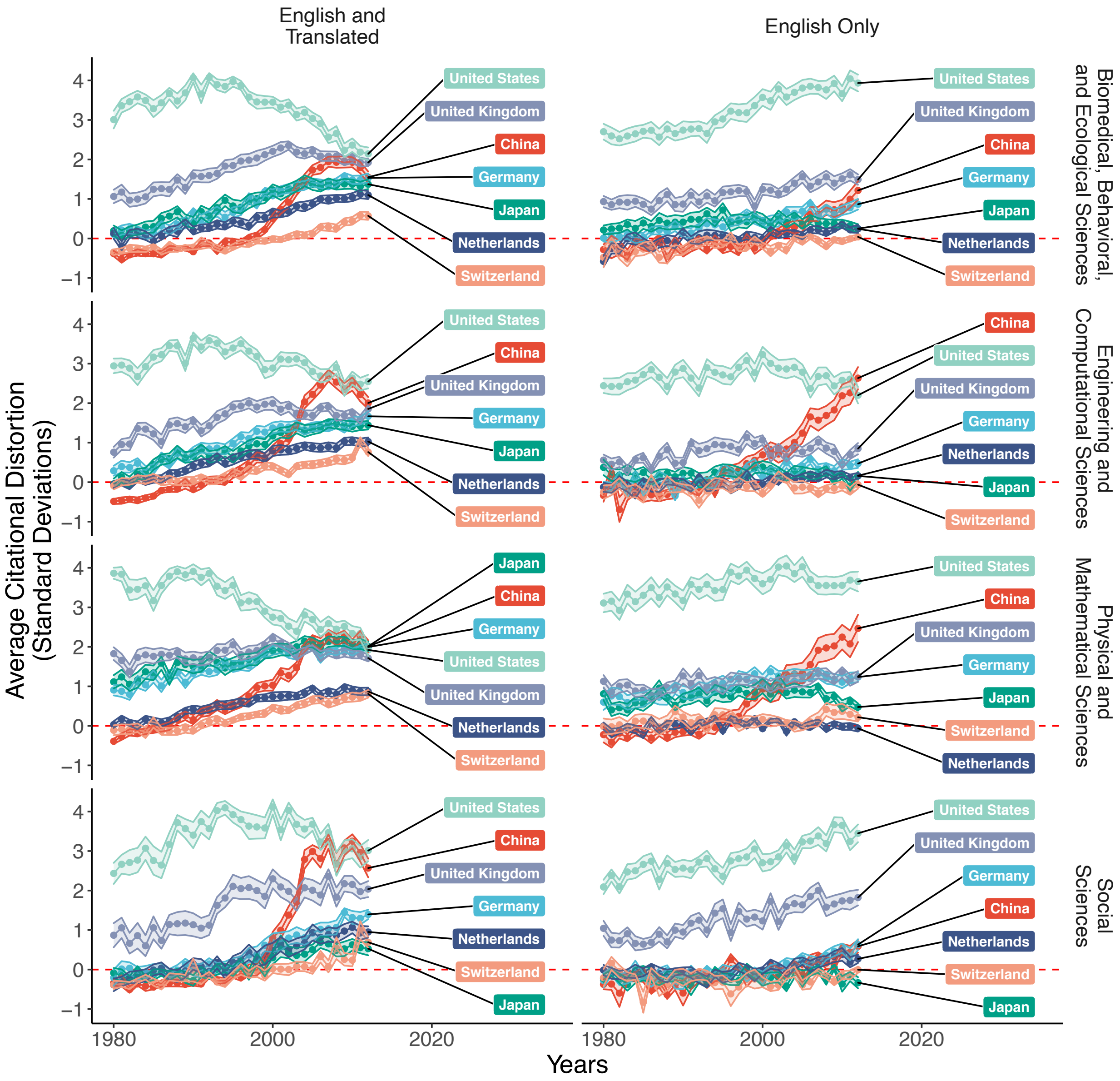

Supplementary Figure 26. Average citational distortion for countries comparing English censored abstracts over time by research area. Plots the trends in Supplementary Figure 24 but parsed by the type of field. We include two panes: one where the networks were built from English and translated abstracts and one where the networks used English abstracts only. The shading around the trends denotes the standard errors to these averages.

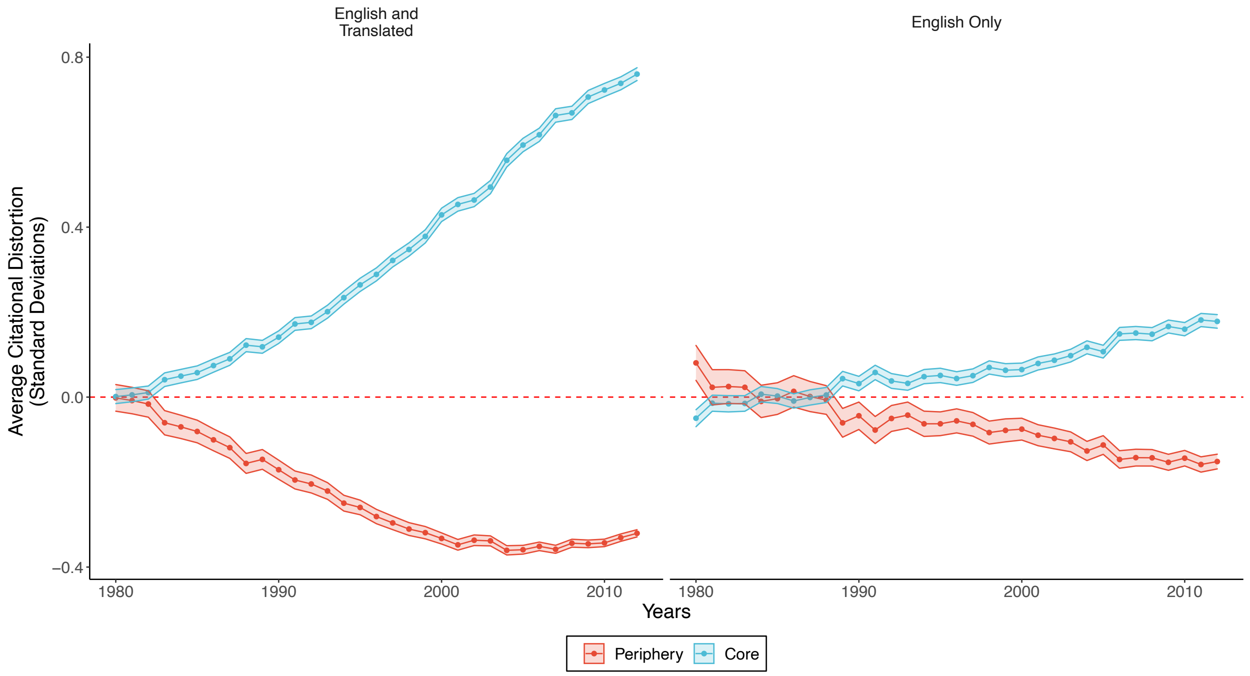

Supplementary Figure 27. Average citational distortion for core and periphery countries comparing English censored abstracts over time by research area. Plots the trends in Supplementary Figure 25 but parsed by the type of field. We include two panes: one where the networks were built from English and translated abstracts and one where the networks used English abstracts only. The shading around the trends denotes the standard errors to these averages.

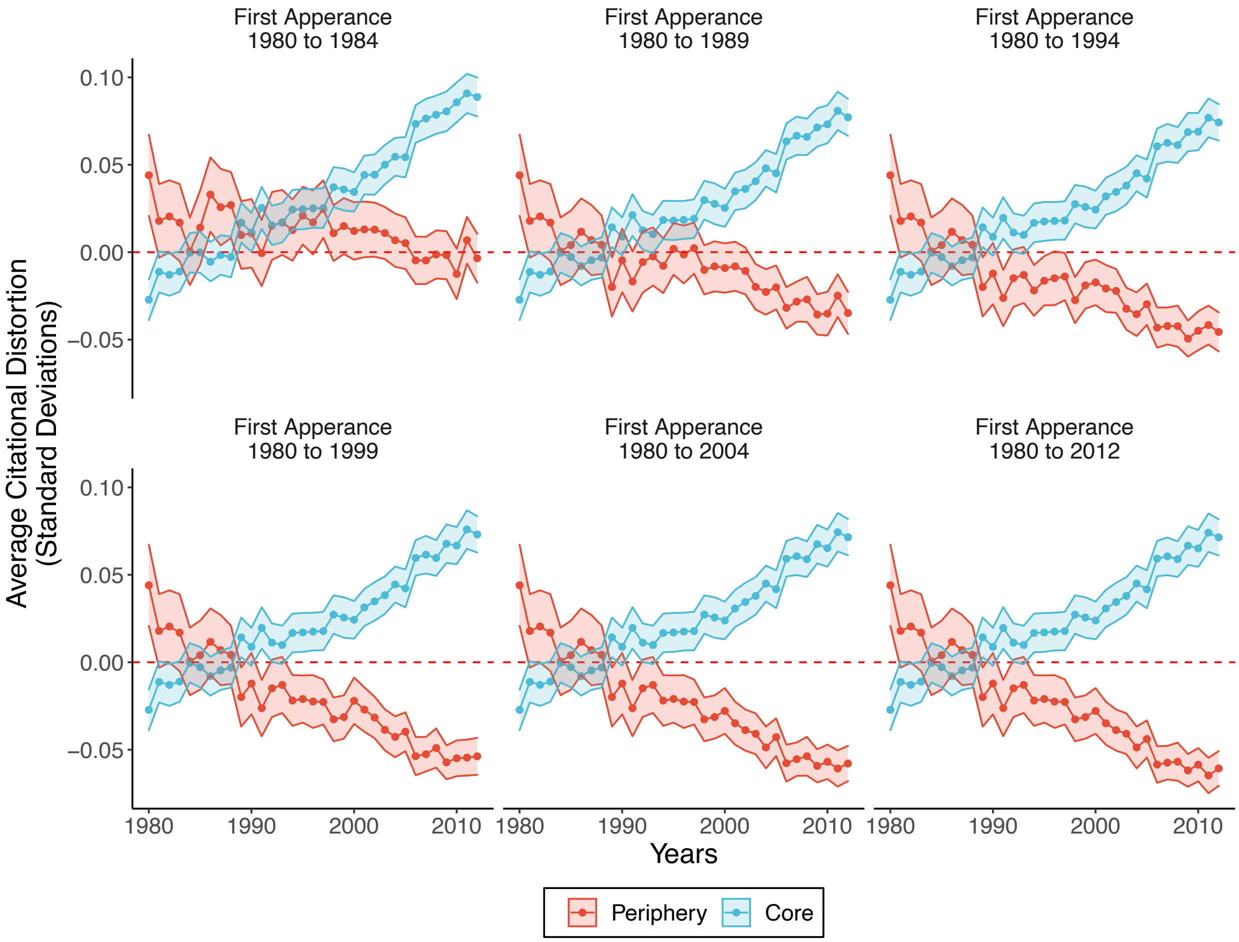

Supplementary Figure 28. Average citational distortion for core and periphery countries by the first appearance of countries in each field. We censor the indegree centrality trends between core and periphery countries based on when the country first appeared in the data in each field. Once the countries are censored, we recalculate the average across fields. We include six panes, one for each time window that a country appeared in the data for each field. The shading around the trends denotes the standard errors to these averages.

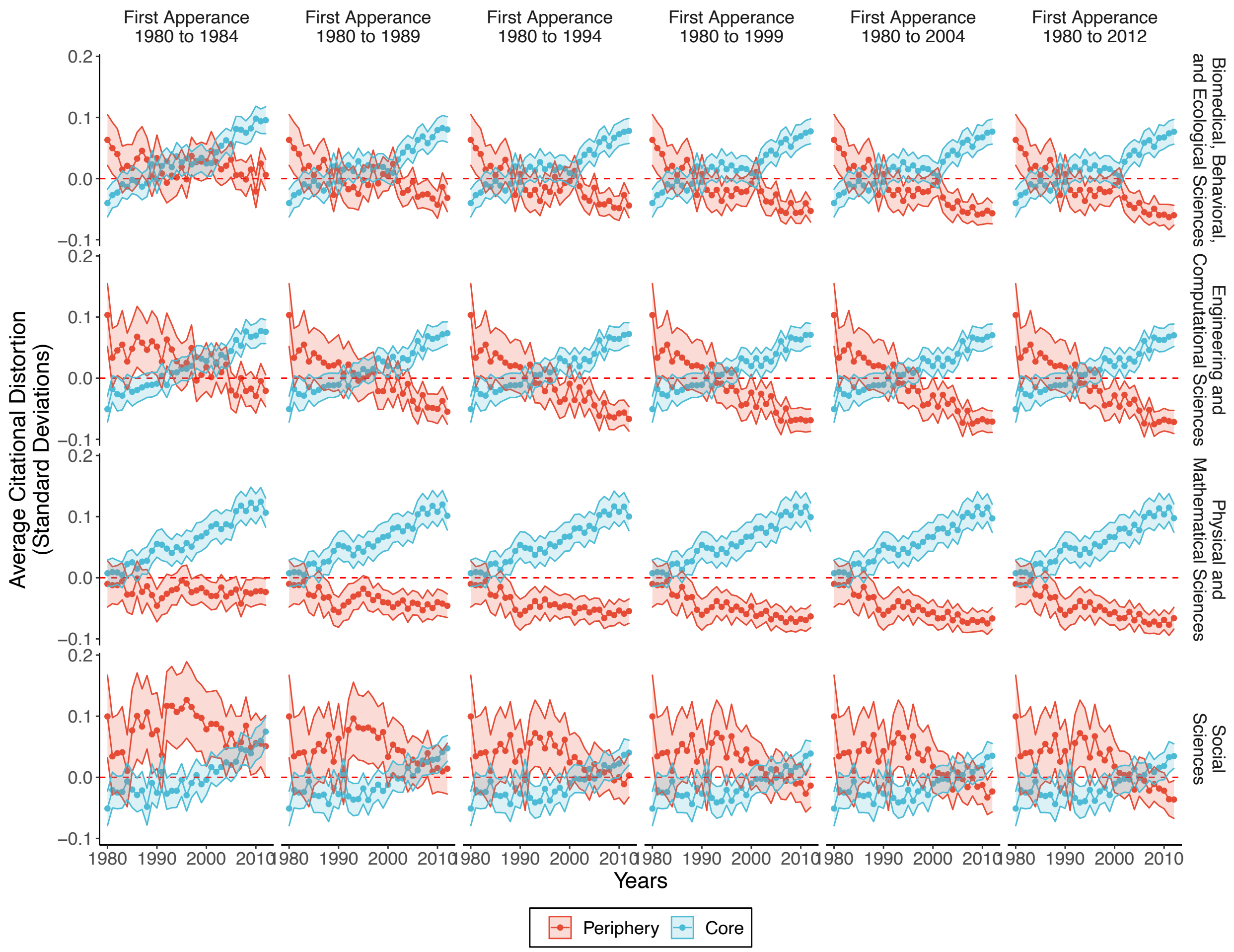

Supplementary Figure 29. Average citational distortion for core and periphery countries by the first appearance of countries in each field. Plots the trends in Supplementary Figure 28 but parsed by the type of field. We include six panes, one for each time window that a country appeared in the data for each field. The shading around the trends denotes the standard errors to these averages.

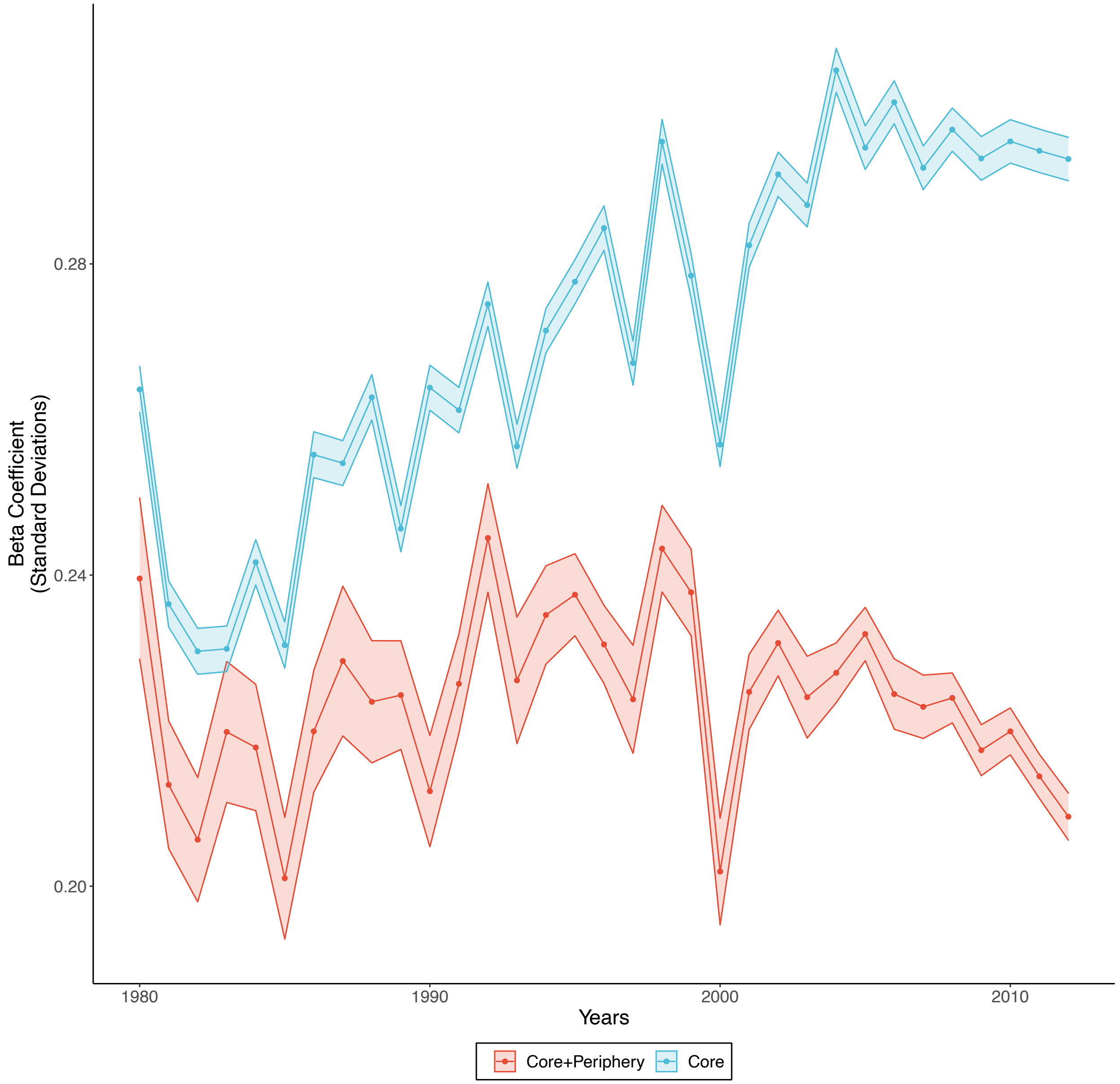

Supplementary Figure 30. Relationship between citations and text that includes both statistically-significant and not statistically-significant beta coefficients. We plot the average of beta coefficients that includes both statistically-significant and not statistically-significant coefficients from each field's yearly QAP model for citations plotted over time from 1980 to 2012. The shading around the trends denotes the grand standard errors across beta-coefficients.

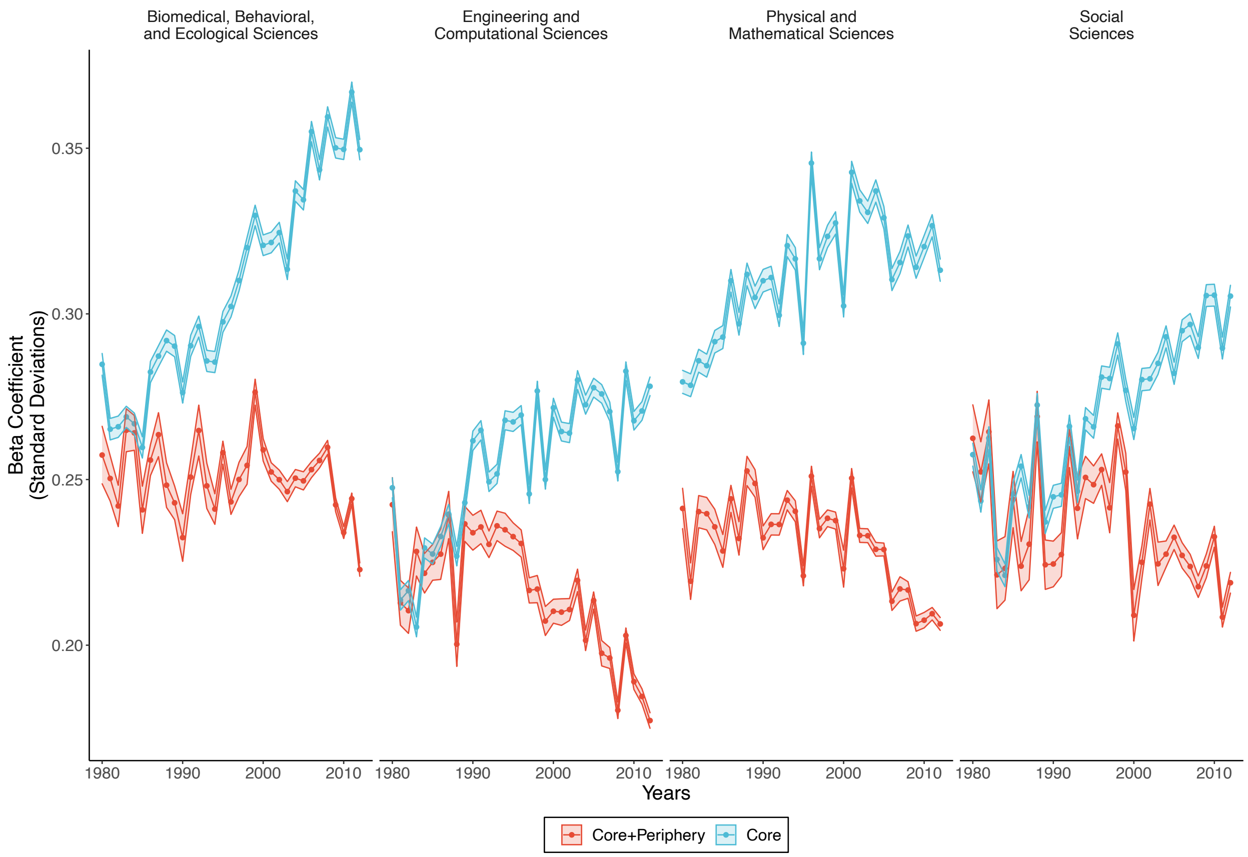

Supplementary Figure 31. Relationship between citations and text that includes both statistically-significant and not statistically-significant beta coefficients by research area. Plots the trends in Supplementary Figure 30 but parsed by the type of field. The shading around the trends denotes the standard errors to these averages.

<table>
<tr><th></th><th>Name</th></tr>
<tr><td>Biomedical, Behavioral, and Ecological Sciences</td><td>Agroforestry | Alternative Medicine | Andrology | Anesthesia | Astrobiology | Audiology | Cancer Research | Cognitive Psychology | Cognitive Science | Computational Biology | Dentistry | Dermatology | Emergency Medicine | Environmental Health | Environmental Science | Evolutionary Biology | Fishery | Forestry | Gastroenterology | Gerontology | Gynecology | Medical Emergency | Medical Physics | Medicinal Chemistry | Nuclear Medicine | Obstetrics | Ophthalmology | Optometry | Orthodontics | Paleontology | Physical Medicine and Rehabilitation | Physiology | Toxicology | Traditional Medicine | Urology | Veterinary Medicine | Zoology</td></tr>
<tr><td>Engineering and Computational Sciences</td><td>Aerospace Engineering | Algorithm | Architectural Engineering | Automotive Engineering | Biomedical Engineering | Biotechnology | Ceramic Materials | Chemical Engineering | Civil Engineering | Computer Engineering | Computer Hardware | Computer Security | Computer Vision | Control Engineering | Crystallography | Data Mining | Data Science | Database | Distributed Computing | Electronic Engineering | Embedded System | Engineering Physics | Environmental Engineering | Environmental Protection | Environmental Resource Management | Forensic Engineering | Geotechnical Engineering | Human-Computer Interaction | Hydrology | Information Retrieval | Manufacturing Engineering | Mechanical Engineering | Nanotechnology | Natural Language Processing | Nuclear Engineering | Parallel Computing | Pattern Recognition | Petroleum Engineering | Programming Language | Reliability Engineering | Software Engineering | Speech Recognition | Systems Engineering | Telecommunications | Theoretical Computer Science | Transport Engineering | Water Resource Management</td></tr>
<tr><td>Physical and Mathematical Sciences</td><td>Astronomy | Astrophysics | Atomic Physics | Biophysics | Chemical Physics | Classical Mechanics | Combinatorics | Computational Physics | Computational Science | Earth Science | Geodesy | Geometry | Geomorphology | Geophysics | Mathematical Physics | Mechanics | Mineralogy | Molecular Physics | Nuclear Chemistry | Nuclear Physics | Oceanography | Petrology | Physical Chemistry | Polymer Chemistry | Quantum Mechanics | Radiochemistry | Seismology | Soil Science | Statistical Physics | Statistics | Theoretical Physics | Thermodynamics</td></tr>
<tr><td>Social Sciences</td><td>Accounting | Actuarial Science | Advertising | Aesthetics | Agricultural Economics | Anthropology | Applied Psychology | Archaeology | Commerce | Communication | Criminology | Demography | Environmental Ethics | Epistemology | Ethnology | Finance | Financial System | Gender Studies | Industrial Organization | Law | Library Science | Linguistics | Management | Management Science | Operations Research | Pedagogy | Philosophy | Physical Geography | Political Economy | Religious Studies | Sociology | Theology</td></tr>
</table>

Supplementary Table 1. Fields by research area. List of fields analyzed parsed into four broad categories: (1) biomedical, behavioral, and ecological sciences; (2) engineering and computational sciences; (3) physical and mathematical sciences; and (4) the social sciences.

<table>
<tr><th></th><th>Name</th></tr>
<tr><td>Core</td><td>Australia | Austria | Belgium | Canada | China | Denmark | Finland | France | Germany | Greece | Greenland | Iceland | Ireland | Israel | Italy | Japan | Liechtenstein | Luxembourg | Monaco | Netherlands | New Zealand | Norway | Portugal | Singapore | South Korea | Spain | Sweden | Switzerland | Taiwan | United Kingdom | United States</td></tr>
<tr><td>Periphery</td><td>Afghanistan | Aland Islands | Albania | Algeria | Antigua and Barbuda | Argentina | Armenia | Aruba | Azerbaijan | Bahamas | Bahrain | Bangladesh | Belarus | Belize | Benin | Bermuda | Bhutan | Bolivia | Bosnia and Herzegovina | Botswana | Brazil | British Virgin Islands | Brunei | Bulgaria | Burkina Faso | Burundi | Cambodia | Cameroon | Cape Verde | Cayman Islands | Chad | Chile | Colombia | Costa Rica | Croatia | Cuba | Curacao | Cyprus | Czechia | Democractic Republic of the Congo | Dominica | Dominican Republic | Ecuador | Egypt | El Salvador | Eritrea | Estonia | Ethiopia | Fiji | French Polynesia | Gabon | Gambia | Georgia | Ghana | Gibraltar | Grenada | Guadeloupe | Guatemala | Guinea | Guinea-Bissau | Guyana | Hungary | India | Indonesia | Iran | Iraq | Ivory Coast | Jamaica | Jordan | Kazakhstan | Kenya | Kosovo | Kuwait | Kyrgyzstan | Laos | Latvia | Lebanon | Lesotho | Liberia | Libya | Lithuania | Macao | Macedonia | Madagascar | Malawi | Malaysia | Maldives | Mali | Malta | Mauritania | Mauritius | Mexico | Moldova | Mongolia | Montenegro | Montserrat | Morocco | Mozambique | Myanmar | Namibia | Nepal | New Caledonia | Nicaragua | Niger | Nigeria | North Korea | Oman | Pakistan | Palestine | Panama | Papua New Guinea | Paraguay | Peru | Philippines | Poland | Qatar | Republic of the Congo | Reunion | Romania | Russia | Saint Kitts and Nevis | Saint Lucia | Saint Vincent | Samoa | Saudi Arabia | Senegal | Serbia | Sierra Leone | Sint Maarten | Slovakia | Slovenia | Somalia | South Africa | South Sudan | Sri Lanka | Sudan | Suriname | Swaziland | Syria | Tajikistan | Tanzania | Thailand | Togo | Trinidad and Tobago | Tunisia | Turkey | Turkmenistan | Uganda | Ukraine | United Arab Emirates | Uruguay | Uzbekistan | Venezuela | Vietnam | Yemen | Zambia | Zimbabwe</td></tr>
</table>

Supplementary Table 2. Countries by Core and Periphery. Note that some countries are disputed, overseas territories, principalities, or other state forms that we collectively refer to as "countries."

	Model 1	Model 2	Model 3	Model 4	Model 5
(Intercept)	-0.07^{***}	-0.07^{***}	-0.06^{***}	-0.04^{***}	-0.04^{***}
	(0.00)	(0.00)	(0.00)	(0.00)	(0.00)
Top 50 Universities		0.02^{***}			-0.01^{***}
		(0.00)			(0.00)
R&D Percentage			0.02^{***}		0.01^{***}
			(0.00)		(0.00)
Number of Papers				0.13^{***}	0.13^{***}
				(0.00)	(0.00)
AIC	77520.59	76787.34	76790.56	64488.75	64119.26
BIC	77560.82	76837.62	76840.84	64539.03	64189.66
Log Likelihood	-38756.29	-38388.67	-38390.28	-32239.37	-32052.63
Num. obs.	172370	172370	172370	172370	172370
Num. groups: Country:Discipline	12711	12711	12711	12711	12711
Num. groups: Discipline	148	148	148	148	148
Var: Country:Discipline (Intercept)	0.10	0.10	0.10	0.05	0.05
Var: Discipline (Intercept)	0.00	0.00	0.00	0.00	0.00
Var: Residual	0.08	0.08	0.08	0.07	0.07

$^{***}p < 0.001$; $^{**}p < 0.01$; $^{*}p < 0.05$

Supplementary Table 3. Hierarchical linear model (HLM) regressing in-degree citational distortion of countries on the number of papers published with at least one author affiliated with these countries.

	Model 1	Model 2	Model 3	Model 4	Model 5
(Intercept)	-0.23^{***}	-0.23^{***}	-0.21^{***}	-0.16^{***}	-0.15^{***}
	(0.01)	(0.01)	(0.01)	(0.00)	(0.00)
Top 50 Universities		0.06^{***}			-0.03^{***}
		(0.00)			(0.00)
R&D Percentage			0.07^{***}		0.05^{***}
			(0.00)		(0.00)
Number of Papers				0.36^{***}	0.36^{***}
				(0.00)	(0.00)
AIC	221802.71	219522.50	218533.70	177071.52	175203.17
BIC	221843.07	219572.95	218584.15	177121.97	175273.80
Log Likelihood	-110897.36	-109756.25	-109261.85	-88530.76	-87594.58
Num. obs.	178044	178044	178044	178044	178044
Num. groups: Country:Discipline	12915	12915	12915	12915	12915
Num. groups: Discipline	148	148	148	148	148
Var: Country:Discipline (Intercept)	0.31	0.28	0.28	0.16	0.16
Var: Discipline (Intercept)	0.00	0.00	0.00	0.00	0.00
Var: Residual	0.17	0.17	0.16	0.13	0.13

$^{***}p < 0.001$; $^{**}p < 0.01$; $^{*}p < 0.05$

Supplementary Table 4. Hierarchical linear model (HLM) regressing in-degree text similarity of countries on the number of papers published with at least one author affiliated with these countries.
