# Supplementary material for: Leading countries in global science increasingly receive more citations than other countries doing similar research
Source: Nat Hum Behav. 2022 May 30;6(7):919–29. doi: 10.1038/s41562-022-01351-5 (PMC9314251; doi:10.1038/s41562-022-01351-5)
Supplement: Supplementary file 2 — Reporting Summary [file 41562_2022_1351_MOESM2_ESM.pdf]

nature research

Corresponding author(s): Charles J. Gomez

Last updated by author(s): Mar 3, 2022

Reporting Summary

Nature Research wishes to improve the reproducibility of the work that we publish. This form provides structure for consistency and transparency in reporting. For further information on Nature Research policies, see our Editorial Policies and the Editorial Policy Checklist.

Statistics

For all statistical analyses, confirm that the following items are present in the figure legend, table legend, main text, or Methods section.

n/a	Confirmed	
☐	☒	The exact sample size (n) for each experimental group/condition, given as a discrete number and unit of measurement
☒	☐	A statement on whether measurements were taken from distinct samples or whether the same sample was measured repeatedly
☐	☒	The statistical test(s) used AND whether they are one- or two-sided<br>*Only common tests should be described solely by name; describe more complex techniques in the Methods section.*
☐	☒	A description of all covariates tested
☐	☒	A description of any assumptions or corrections, such as tests of normality and adjustment for multiple comparisons
☐	☒	A full description of the statistical parameters including central tendency (e.g. means) or other basic estimates (e.g. regression coefficient) AND variation (e.g. standard deviation) or associated estimates of uncertainty (e.g. confidence intervals)
☐	☒	For null hypothesis testing, the test statistic (e.g. F, t, r) with confidence intervals, effect sizes, degrees of freedom and P value noted<br>*Give P values as exact values whenever suitable.*
☒	☐	For Bayesian analysis, information on the choice of priors and Markov chain Monte Carlo settings
☒	☐	For hierarchical and complex designs, identification of the appropriate level for tests and full reporting of outcomes
☐	☒	Estimates of effect sizes (e.g. Cohen's d, Pearson's r), indicating how they were calculated

Our web collection on statistics for biologists contains articles on many of the points above.

Software and code

Policy information about availability of computer code

Data collection	Data are primarily from the publicly available Microsoft Academic Graph (MAG).
Data analysis	Data were analyzed, processed, and visualized using Python 3, R, and SQL. The data are stored on Athena (Amazon), queried and read in to Python, analyzed with Python, and visualized with R.

For manuscripts utilizing custom algorithms or software that are central to the research but not yet described in published literature, software must be made available to editors and reviewers. We strongly encourage code deposition in a community repository (e.g. GitHub). See the Nature Research guidelines for submitting code & software for further information.

Data

Policy information about availability of data

All manuscripts must include a data availability statement. This statement should provide the following information, where applicable:

- Accession codes, unique identifiers, or web links for publicly available datasets
- A list of figures that have associated raw data
- A description of any restrictions on data availability

All data derived from the Microsoft Academic Graph (e.g., corpora, NL-LDA models, CSV edgelists, etc.), along with all relevant code in Python 3 and R used to analyze and visualize these data, will be made available at the Harvard Dataverse upon publication.

Field-specific reporting

Please select the one below that is the best fit for your research. If you are not sure, read the appropriate sections before making your selection.

☐ Life sciences ☒ Behavioural & social sciences ☐ Ecological, evolutionary & environmental sciences

For a reference copy of the document with all sections, see nature.com/documents/nr-reporting-summary-flat.pdf

Behavioural & social sciences study design

All studies must disclose on these points even when the disclosure is negative.

Study description	The data are quantitative and text-based. The text data were processed using nation-labeled LDA models and using MRQAP network regression models.
Research sample	We selected 181 field IDs from the Microsoft Academic Graph (MAG) and read in all of the published paper metadata from papers published between 1980 and 2015, inclusive.
Sampling strategy	No sample size calculations were performed. Fields in MAG are classified into a several-tier hierarchy that progresses deeper into field sub-specialties. We selected fields from tier-1 which has the most representative set of fields without selecting from sub-specialties.
Data collection	Data were procured from the publicly available Microsoft Academic Graph (MAG).
Timing	Our metadata in the Microsoft Academic Graph (MAG) extend to 2017. We stop our analyses in 2015 since the total number of papers in 2017 drops compared to 2016, indicating that these data may not be fully complete.
Data exclusions	Only papers that had abstract text data were used in our analyses.
Non-participation	Participants were not used in this study.
Randomization	Randomization strategies were not relevant to this study.

Reporting for specific materials, systems and methods

We require information from authors about some types of materials, experimental systems and methods used in many studies. Here, indicate whether each material, system or method listed is relevant to your study. If you are not sure if a list item applies to your research, read the appropriate section before selecting a response.

Materials & experimental systems

n/a	Involved in the study	
☒	☐	Antibodies
☒	☐	Eukaryotic cell lines
☒	☐	Palaeontology and archaeology
☒	☐	Animals and other organisms
☒	☐	Human research participants
☒	☐	Clinical data
☒	☐	Dual use research of concern

Methods

n/a	Involved in the study	
☒	☐	ChIP-seq
☒	☐	Flow cytometry
☒	☐	MRI-based neuroimaging
